# Mutagenesis of *GmFT2a* and *GmFT5a* mediated by CRISPR/Cas9 contributes for expanding the regional adaptability of soybean

**DOI:** 10.1111/pbi.13199

**Published:** 2019-07-05

**Authors:** Yupeng Cai, Liwei Wang, Li Chen, Tingting Wu, Luping Liu, Shi Sun, Cunxiang Wu, Weiwei Yao, Bingjun Jiang, Shan Yuan, Tianfu Han, Wensheng Hou

**Affiliations:** ^1^ National Center for Transgenic Research in Plants Institute of Crop Sciences Chinese Academy of Agricultural Sciences Beijing China; ^2^ Ministry of Agriculture Key Laboratory of Soybean Biology (Beijing) Institute of Crop Sciences Chinese Academy of Agricultural Sciences Beijing China

**Keywords:** soybean, CRISPR/Cas9, *GmFT2a*, *GmFT5a*, flowering time, regional adaptability

## Abstract

Flowering time is a key agronomic trait that directly influences the successful adaptation of soybean (*Glycine max*) to diverse latitudes and farming systems. *GmFT2a* and *GmFT5a* have been extensively identified as flowering activators and integrators in soybean. Here, we identified two quantitative trait loci (QTLs) regions harbouring *GmFT2a* and *GmFT5a*, respectively, associated with different genetic effects on flowering under different photoperiods. We analysed the flowering time of transgenic plants overexpressing *GmFT2a* or *GmFT5a*,* ft2a* mutants, *ft5a* mutants and *ft2aft5a* double mutants under long‐day (LD) and short‐day (SD) conditions. We confirmed that *GmFT2a* and *GmFT5a* are not redundant, they collectively regulate flowering time, and the effect of *GmFT2a* is more prominent than that of *GmFT5a* under SD conditions whereas *GmFT5a* has more significant effects than *GmFT2a* under LD conditions. *GmFT5a*, not *GmFT2a*, was essential for soybean to adapt to high latitude regions. The *ft2aft5a* double mutants showed late flowering by about 31.3 days under SD conditions and produced significantly increased numbers of pods and seeds per plant compared to the wild type. We speculate that these mutants may have enormous yield potential for the tropics. In addition, we examined the sequences of these two loci in 202 soybean accessions and investigated the flowering phenotypes, geographical distributions and maturity groups within major haplotypes. These results will contribute to soybean breeding and regional adaptability.

## Introduction

Soybean (*Glycine max* (L.) Merr.) originated in the temperate regions of China between 32.0°N and 40.5°N (Li *et al*., 2008b), and has been introduced as a crop plant to Korea, Japan and many countries in North/Central/South America. It has become one of the most important economic crops on account of its high oil and protein concentrations (Wilson, 2008). A major factor for the distribution of soybean cultivation across a wide range of geographical regions, and responsible for large impacts on yield, is its diversity in flowering time. Floral transition is synchronized by the integration of signals from factors that are both endogenous (such as gene, plant age and hormone status) and environmental (such as photoperiod and temperature) (Song *et al*., 2013). Among the various environmental signals, photoperiod is one of the major determinants of soybean's adaptation to seasonal changes in day length for flowering (Bäurle and Dean, 2006). Soybean is known as a short‐day plant that accelerates transition from the vegetative phase to the reproductive phase when it senses short‐day (SD) conditions. Soybean still flowers under long‐day (LD) conditions, albeit much later than in SD conditions. Thus, investigating the responses of flowering to photoperiod has great significance in soybean regional introduction and domestication (Wang *et al*., 2016).

In the model plant *Arabidopsis thaliana*, at least four major flowering pathways, namely the vernalization, autonomous, gibberellin (GA) and photoperiod pathways, are known to regulate the floral transition process (Fornara *et al*., 2010). These four pathways regulate the expression of *FLOWERLOCUST* (*FT*), which plays an important role in flowering pathways as an integrator (Turck *et al*., 2008) and encodes a florigen protein that is transported from leaves to shoot apical meristems (SAMs) through the phloem and that functions as a long‐distance signal to induce floral initiation (Corbesier *et al*., 2007; Jaeger and Wigge, 2007; Mathieu *et al*., 2007; Notaguchi *et al*., 2008). FT and FD (a bZIP transcription factor) are interdependent partners through protein interaction and act at the SAMs to promote floral transition through transcriptional activation of the floral meristem identity gene *APETALA1* (*AP1*) (Abe *et al*., 2005; Wigge *et al*., 2005). Ectopic expression of *ArabidopsisFT* in soybean induced precocious flowering under non‐inductive conditions (Yamagishi and Yoshikawa, 2011), providing evidence that *FT* promotes flowering in soybean as well.

Ten *FT* homologs have been identified in soybean. Among them, *GmFT2a* (Glyma16g26660) and *GmFT5a* (Glyma16g04830) have been confirmed to have main promoting effects on flowering time (Kong *et al*., 2010). At present, there are three models, *PHYA*‐*E1*,* GI*‐*CO* and microRNA‐dependent modules that are known to regulate flowering time in soybean. *E1* is a soybean‐specific transcription factor, which is significantly suppressed under SD conditions but is induced under LD conditions. The expression of *E1* is controlled by two *phytochromeA* (*PHYA*) homologs, *E3* (Glyma19g41210) (Watanabe *et al*., 2009) and *E4* (Glyma20g22160) (Liu *et al*., 2008), and exhibits a bimodal pattern at dawn and dusk under LD conditions (Xia *et al*., 2012). *J*, as the ortholog of *Arabidopsis thaliana EARLYFLOWERING3* (*ELF3*) (Yue *et al*., 2017), acts upstream of *E1* in the soybean flowering pathway and down‐regulates *E1* transcription to relieve repression of *GmFT2a* and *GmFT5a* under SD conditions. In addition, expression of *J* is suppressed by the combined action of *E3* and *E4* under SD conditions (Lu *et al*., 2017).

*GmFT2a* and *GmFT5a* are up‐regulated under SD conditions, whereas they are significantly down‐regulated under LD conditions (Kong *et al*., 2010; Xu *et al*., 2013). Interestingly, a previous SD‐to‐LD transfer experiments showed that *GmFT2a* expression was more sensitive to photoperiodic changes than that of *GmFT5a* (Kong *et al*., 2010), and it is worth noting that only the *GmFT5a* transcripts accumulated in the late growth stage of soybean under LD conditions. These results indicated that, in addition to *PHYA*‐mediated flowering pathways, *GmFT5a* may be controlled in a photoperiod‐independent manner by another regulatory mechanism in longer day lengths (Kong *et al*., 2010). An ortholog of *Arabidopsis thaliana GIGANTEA* (*GI*), *GmGIa* (Glyma10g36600), is responsible for the *E2* locus. Under natural day length conditions, *E2* inhibits the expression of *GmFT2a*, whereas the expression level of *GmFT5a* is not controlled by the *E2* locus (Watanabe *et al*., 2011).

Under LD conditions, the expression of *GmmiR156b*, a flowering suppressor in soybean, is up‐regulated by *E1*,* E2*,* E3*, and *E4*, and can suppress *E1* (*E1‐Like*) and *E2* (*E2‐Like*) genes. Overexpression of *GmmiR156b* caused a significantly late‐flowering phenotype under LD conditions, whereas it caused a slightly late flowering under SD conditions. Importantly, the expression level of *GmFT5a* is down‐regulated by the overexpression of *GmmiR156b* under LD or SD conditions, whereas *GmFT2a* is decreased only under SD conditions (Cao *et al*., 2015). Both GmFT2a and GmFT5a proteins interact with the bZIP transcription factor GmFDL19 in soybean and then up‐regulate several downstream flowering‐related genes (such as *GmAP1*,* GmSOC1* and *GmLFY*) in both a redundant and differential pattern, suggesting that the FT/FD‐AP1 module is conserved in soybean (Nan *et al*., 2014). Overexpression of *GmFT2a* or *GmFT5a* in the soybean variety Williams 82 promotes early flowering under LD conditions (Nan *et al*., 2014).

The soybean *E9* has been confirmed as *GmFT2a*, and the transcriptional expression level of its recessive allele *e9* (*E9* and *e9* contain the same coding sequence) is always lower than that of *E9* under any photoperiod conditions, and this results from a *Ty1/copia‐like* retrotransposon *SORE‐1* that is highly methylated and inserted in the first intron of *e9*, thus cause late‐flowering phenotype (Zhao *et al*., 2016). In a previous study, we employed the CRISPR/Cas9 system to specifically induce targeted mutagenesis of *GmFT2a* in the soybean variety Jack. Site‐directed mutations in the first exon of *GmFT2a* generated frameshift mutations and then damaged the gene functions. The homozygous *ft2a* mutants exhibited late‐flowering phenotype under both LD and SD conditions (Cai *et al*., 2018).

*GmFT2a* and *GmFT5a* often appear in pairs as flowering promoters in previous studies. However, information about the individual effects of *GmFT2a* and *GmFT5a* in floral induction and their differential regulation of downstream flowering‐related genes under SD and LD conditions remain limited. In this study, we created transgenic plants overexpressing *GmFT2a* or *GmFT5a* (*GmFT2a*‐ox plants, *GmFT5a*‐ox plants), *ft2a* mutants, *ft5a* mutants and *ft2aft5a* double mutants to dissect individual effects of *GmFT2a* and *GmFT5a* on flowering time under LD and SD conditions.

## Results

### Genetic effects of *GmFT2a* and *GmFT5a* on flowering time under different photoperiods

**Figure 1:**
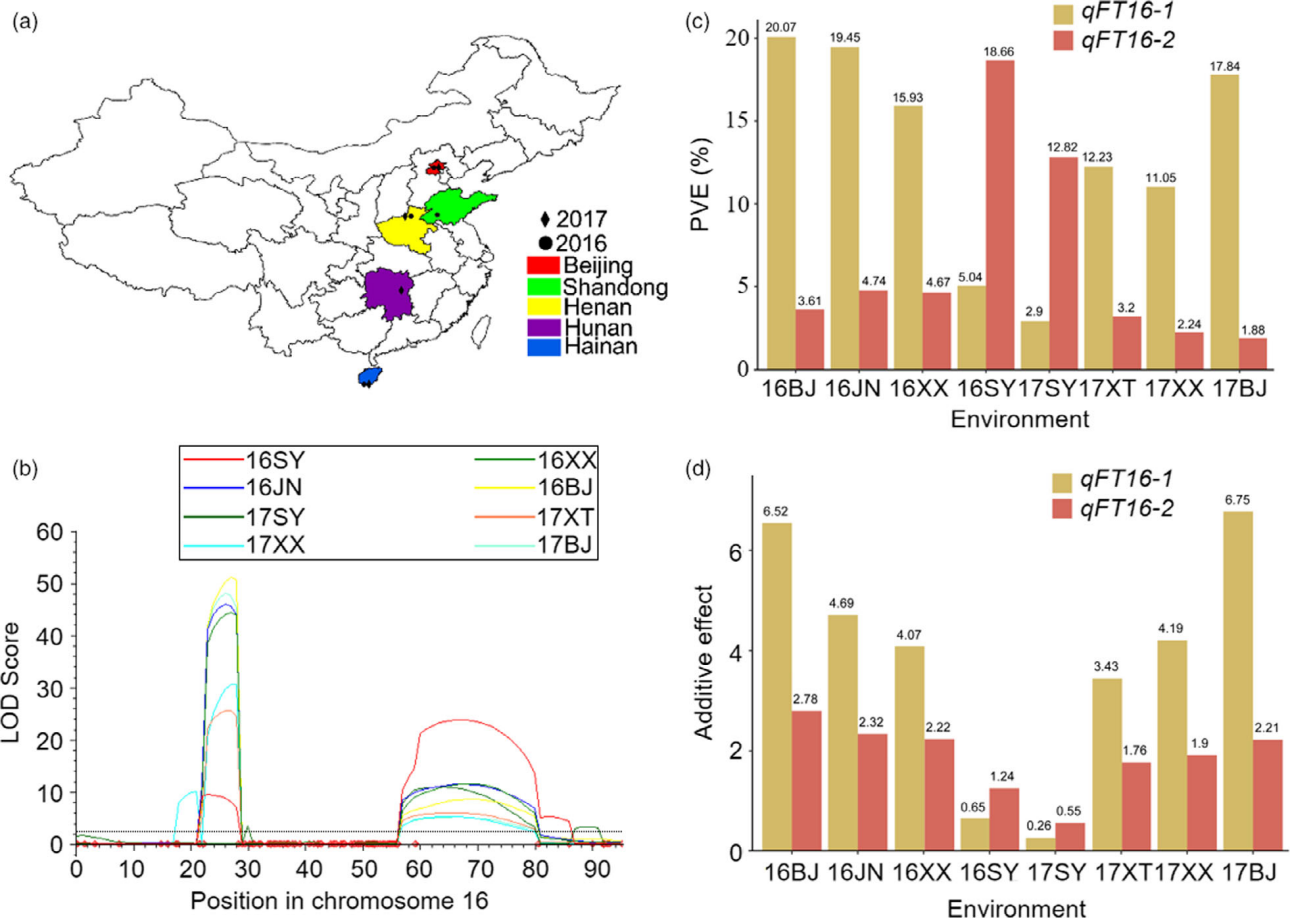
Genetic effects of *qFT16‐1* and *qFT16‐2* under different photoperiods. The eight environments: Beijing on 3 July 2016 and 17 June 2017, Xinxiang on 5 July 2016 and 22 June 2017, Sanya on 19 December 2016 and 18 March 2017, Jining on 3 July 2016, and Xiangtan on 20 June 2017, were named 16BJ, 17BJ, 16XX, 17XX, 16SY, 17SY, 16JN and 17XT, respectively. (a) Geographic distribution of these environments in China. (b) The dotted line represents the LOD (likelihood of odd) score = 3.2. (c) PVE of *qFT16‐1* and *qFT16‐2* in the eight environments. (d) Additive effects of *qFT16‐1* and *qFT16‐2* in the eight environments.

The soybean variety ZGDD showed extremely late flowering under LD conditions, but not under SD conditions, whereas variety HH27 showed early flowering under LD and SD conditions. The 308 F_6:7_ RILs (recombination inbred lines) developed from crosses between ZGDD and HH27 as the parental lines were planted with two replicates in randomized complete blocks in Beijing (N40°13′,E116°33′) on 3 July 2016 and 17 June 2017, Xinxiang (N35°08′, E113°45′) on 5 July 2016 and 22 June 2017, Sanya (N18°18′, E109°30′) on 19 December 2016 and 18 March 2017, Jining (N35°27′, E116°34′) on 3 July 2016 and Xiangtan (N27°40′, E112°39′) on 20 June 2017, and these eight environments were named 16BJ, 17BJ, 16XX, 17XX, 16SY, 17SY, 16JN and 17XT, respectively (Figure 1a). The 308 RIL population was genotyped, and a high‐density genetic map with a total length of 2208.16 cm, comprising 20 linkage groups with an average interval between adjacent markers of 0.64 cm, was constructed.

Based on the genetic map, two major effect QTLs for flowering time—*qFT16‐1* and *qFT16‐2*, both on chromosome 16—were identified across all eight environments (Figure 1b and Table S1). The major QTL *qFT16‐1* explained 2.9%–20.07% of the phenotypic variance. The other major QTL, *qFT16‐2*, accounted for 1.88%–18.66% of the phenotypic variation. To validate our QTL mapping results, we compared the stable QTL *qFT16‐1* and *qFT16‐2* with the previously reported QTLs and genes involved in soybean flowering time regulation; two major previously reported flowering accelerator (Kong *et al*., 2010; Takeshima *et al*., 2016; Zhao *et al*., 2016) genes (*GmFT5a* and *GmFT2a*) are located, respectively, in the genomic regions wherein we detected *qFT16‐1* and *qFT16‐2*. We found no frameshifts or premature stop codons in the coding regions of *GmFT2a* or *GmFT5a* in HH27 and ZGDD (Appendix S1). Although the *GmFT5a* coding regions were identical, Takeshima *et al*. (2016) detected 15 DNA polymorphisms between parents with the early‐flowering and late‐flowering alleles in the promoter region, an intron, and the 3′ untranslated region of *GmFT5a*, and confirmed that *GmFT5a* is the gene responsible for *qDTF‐J1*. Sun *et al*. (2011) demonstrated that the expression of *GmFT2a* in HH27 is higher than that in ZGDD under LD and SD photoperiods. Thus, *GmFT5a* and *GmFT2a* are the most likely candidate genes underlying the *qFT16‐1* and *qFT16‐2* QTLs we identified. Interestingly, compared with *qFT16‐2*, the additive effect and the PVE value of *qFT16‐1* was much larger under the six environments with relatively longer day lengths (16BJ, 17BJ, 16XX, 17XX, 16JN and 17XT) but were substantially lower for the two environments with SD conditions (16SY and 17SY) (Figure 1c,d and Figure S1). These results indicate that *GmFT5a* and *GmFT2a* apparently exert distinct genetic effects under different photoperiods.

### *GmFT2a* and *GmFT5a* have different effects on flowering time under SD and LD conditions

**Figure 2:**
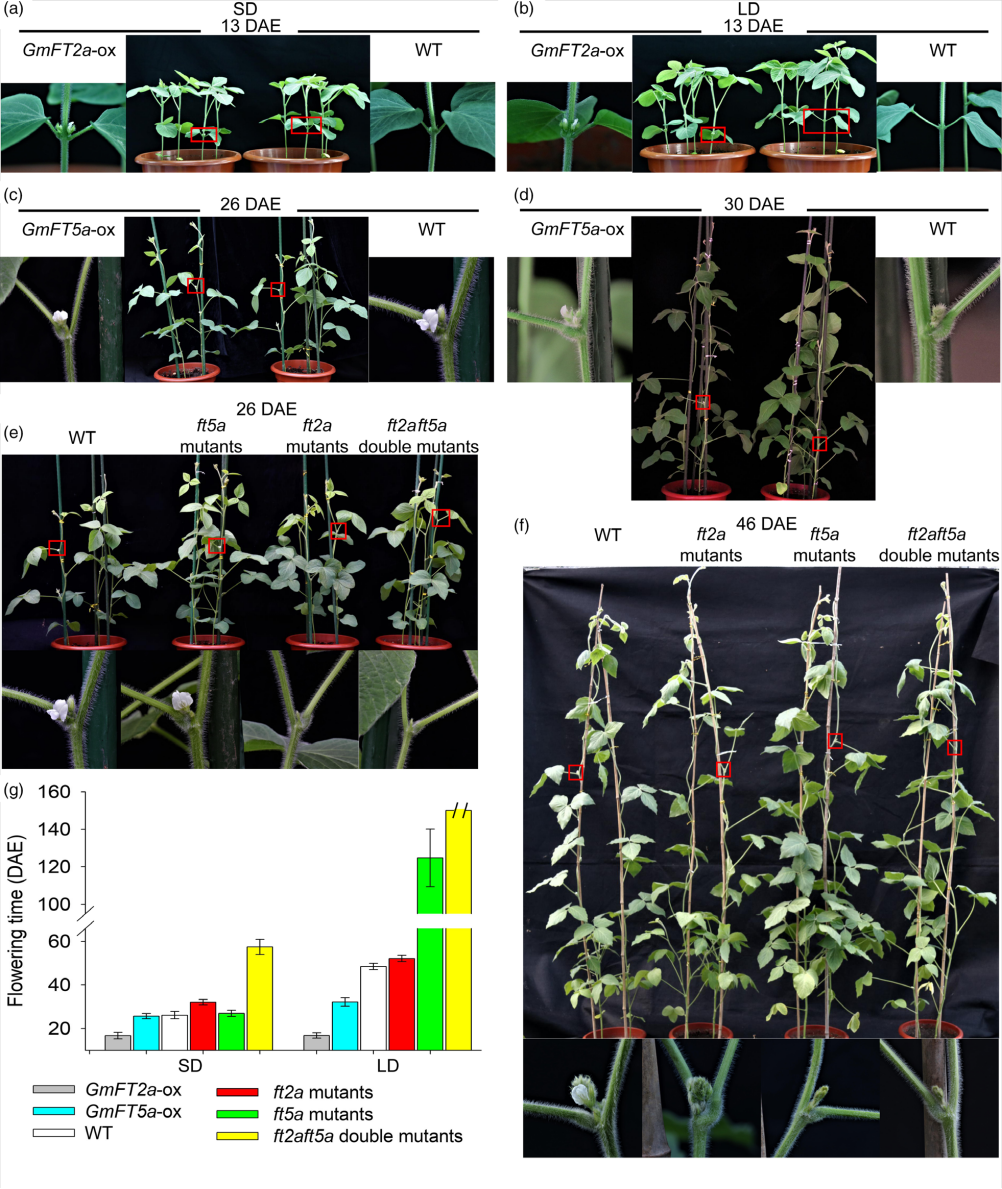
(a) Flowering time of WT plants and *GmFT2a*‐ox plants under SD conditions. (b) Flowering time of WT plants and *GmFT2a*‐ox plants under LD conditions. (c) Flowering time of WT plants and *GmFT5a*‐ox plants under SD conditions. (d) Flowering time of WT plants and *GmFT5a*‐ox plants under LD conditions. (e) Flowering time of WT plants, *ft2a* mutants, *ft5a* mutants, and *ft2aft5a* double mutants under SD conditions. (f) Flowering time of WT plants, *ft2a* mutants, *ft5a* mutants, and *ft2aft5a* double mutants under LD conditions.

Combining the aforementioned results, we suggest that *GmFT5a* and *GmFT2a* have different genetic effects on flowering time under different photoperiods: *GmFT5a* has more effects than *GmFT2a* in higher latitudes. To further evaluate this hypothesis, we examined the flowering times of WT plants, *GmFT2a*‐ox plants, *GmFT5a*‐ox plants, *ft2a* mutants, *ft5a* mutants and *ft2aft5a* double mutants. Under SD (12 h light/12 h dark) conditions and compared to wild‐type (WT) plants, the *GmFT2a*‐ox plants displayed early flowering by about 9 days and the *ft2a* mutants exhibited late flowering by about 6 days (16.7 ± 1.5 DAE for *GmFT2a*‐ox plants vs. 32.0 ± 1.3 DAE for *ft2a* mutants vs. 26.1 ± 1.7 DAE for WT) (Figure 2a,e,g). The flowering time of *GmFT5a*‐ox plants and *ft5a* mutants was almost the same as WT plants (25.6 ± 1.2 DAE for *GmFT5a*‐ox plants vs. 26.9 ± 1.4 DAE for *ft5a* mutants vs. 26.1 ± 1.7 DAE for WT) (Figure 2c,e,g). The *ft2aft5a* double mutants flowered at 57.4 ± 3.5 DAE, about 31 days later than WT plants (Figure 2e,g).

Under LD (16 h light/8 h dark) conditions and compared to WT plants, the *GmFT2a*‐ox plants showed extremely early‐flowering phenotypes of about 32 days, while the *ft2a* mutants exhibited late flowering by about 3.6 days (16.8 ± 1.2 DAE for *GmFT2a*‐ox plants vs. 52.1 ± 1.5 DAE for *ft2a* mutants vs. 48.5 ± 1.4 DAE for WT) (Figure 2b,f,g). The flowering times of the *GmFT2a*‐ox plants were basically same between SD and LD conditions, suggesting that the overexpression of *GmFT2a* promotes precocious flowering independent of the photoperiod. Unlike the observation of no significant phenotypic alterations in flowering time that we observed under SD conditions, we found that overexpression of *GmFT5a* caused early flowering by about 16 days (32.2 ± 1.9 DAE for *GmFT5a*‐ox plants vs. 48.5 ± 1.4 DAE for WT) under LD conditions (Figure 2d,g). Even more remarkably, the *ft5a* mutants flowered at 124.7 ± 15.4 DAE, about 76 days later than WT plants (Figure 2f,g). The *ft2aft5a* double mutants did not flower up to 150 DAE (Figure 2g).

Synchronously, as shown in Figure S2, under SD13‐LD conditions (shifted to LD after 13 days of SD treatment), the flowering time of WT plants was 30.2 ± 1.0 DAE, about 4 days later than under SD and 18 days earlier than under LD. These results indicate that the activation of flowering as induced by SD conditions can promote flowering even under non‐inductive conditions (LD). The *ft2a* mutants flowered at 37.2 ± 2.0 DAE, exhibiting late flowering by about 7 days compared to WT plants (SD13‐LD). And, more remarkably, the *ft5a* mutants (SD13‐LD) had the same extreme late‐flowering phenotype (126.4 ± 13.5 DAE) as they did under LD conditions (124.7 ± 15.4 DAE). That is to say, the activation of flowering as induced by *GmFT2a* under SD conditions could not completely make up for the inactivation of *GmFT5a* gene under LD conditions. Together, we conclude that *GmFT2a* and *GmFT5a* collectively regulate flowering time in soybean, but the effect of *GmFT2a* is apparently more prominent than that of *GmFT5a* under SD conditions. Conversely, the effect of *GmFT5a* is apparently more prominent than that of *GmFT2a* under LD conditions. Thus, the flowering regulation pathways which include *GmFT2a* and *GmFT5a* as participants are different under SD and LD conditions.

### The effect of *GmFT5a* is dependent on day length in flowering time regulation

Because the *ft5a* mutants exhibited extreme late‐flowering phenotype under LD conditions, but flowered similar to the WT plants under SD conditions, we assumed that the different effects of *GmFT5a* on flowering time regulation may require a critical photoperiod to transform. To test the idea, the WT plants, *ft2a* mutants and *ft5a* mutants were grown under 14H (14 h light/10 h dark) and 15H (15 h light/9 h dark) photoperiodic conditions. As shown in Figure S3, under 14H conditions, the *ft2a* mutants exhibited late flowering by about 3.6 days compared to WT plants (33.6 ± 1.2 DAE for *ft2a* mutants vs. 30.0 ± 0.5 DAE for WT), and the flowering time of *ft5a* mutants was a slightly but significantly later than that of WT plants (31.1 ± 0.5 DAE for *ft5a* mutants vs. 30.0 ± 0.5 DAE for WT). Under 15H conditions, the *ft2a* mutants also exhibited late flowering by about 3.7 days compared to WT plants (34.9 ± 0.8 DAE for *ft2a* mutants vs. 31.2 ± 1.2 DAE for WT), and the *ft5a* mutants exhibited late‐flowering phenotype by about 6.8 days compared to WT plants (38.0 ± 1.6 DAE for *ft5a* mutants vs. 31.2 ± 1.2 DAE for WT).

We summarized the data of flowering time under SD, 14H, 15H and LD conditions and found that the flowering time of WT plants was delayed gradually as day length extended from 12 h to 15 h, but was then delayed significantly under 16 h light/8 h dark conditions. We therefore suggest that the critical photoperiod of soybean variety Jack occurs between 15 h and 16 h. As the day length gradually increased, the degree of the late‐flowering phenotype of the *ft5a* mutants became more and more significant. Intriguingly, the *ft5a* mutants exhibited extreme late‐flowering phenotype under LD conditions, about 76 days later than that of WT plants. These results indicated that the flowering regulation pathways in soybean changed with photoperiod from SD to LD conditions. There is apparently an important flowering regulation pathway that enables short‐day plant soybean adapt to LD conditions, and *GmFT5a* but not *GmFT2a* is apparently essential for this pathway.

### The *ft2aft5a* double mutants showed late flowering and produced more pods and seeds under SD conditions

**Figure 3:**
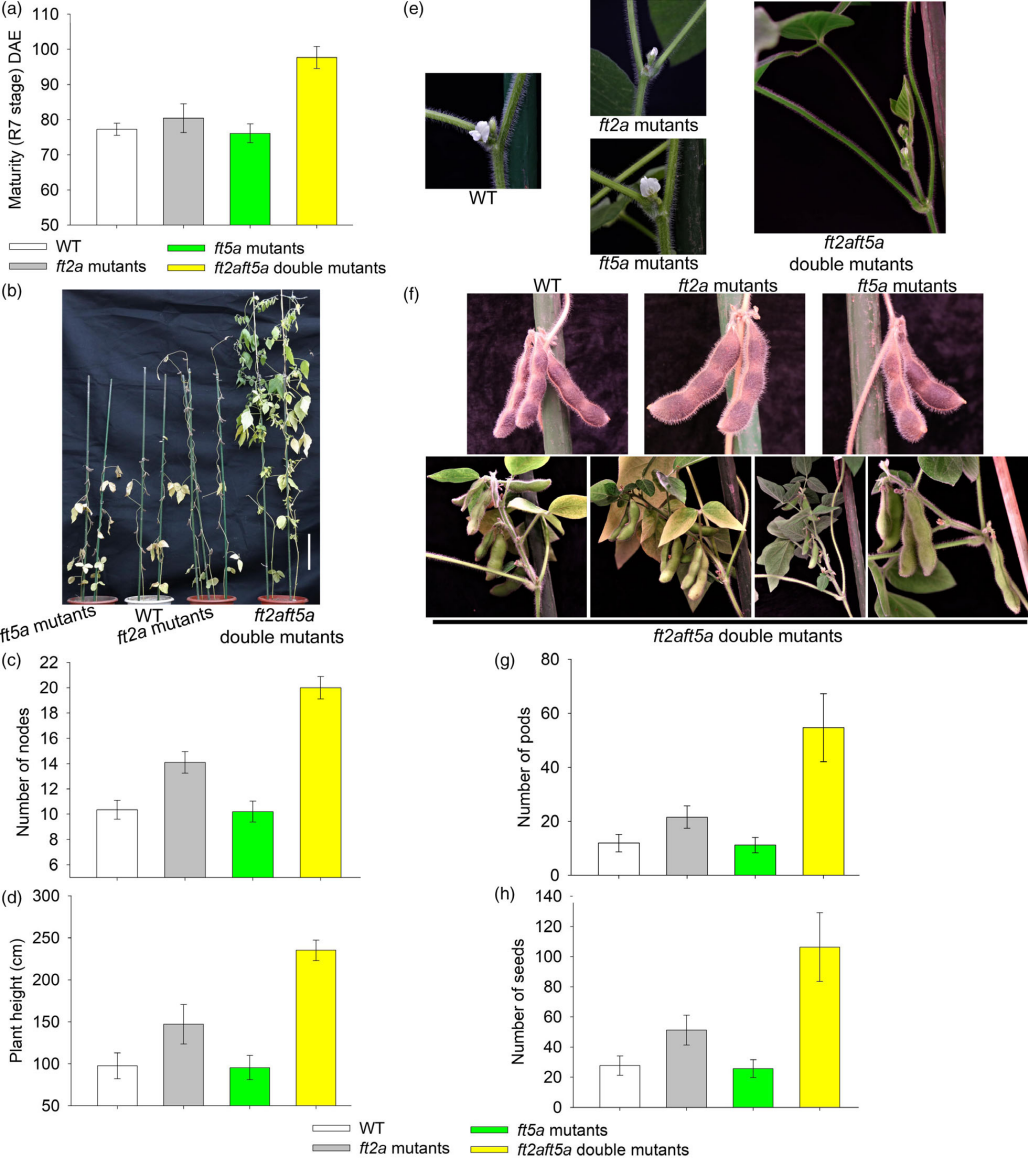
Growth of the WT plants, *ft2a* mutants, *ft5a* mutants and *ft2aft5a* double mutants under SD (12 h light/12 h dark) conditions. (a) Maturity (R7 stage) is shown as the mean values ± standard deviation. DAE, days after emergence. (b) Scale bars, 20 cm. (c) and (d) Number of nodes and plant height are shown as the mean values ± one standard deviation. (e) Flower stalks of WT plants, *ft2a* mutants and *ft5a* mutants located directly at the leaf axil. In contrast, most nodes of the *ft2aft5a* double mutants first produced many new branches instead of flowers at the leaf axil and then produced flower buds at these new branches. (f) New branches at the leaf axil of the *ft2aft5a* double mutants produced more pods and seeds. (g) and (h) Number of pods and seeds are shown as the mean values ± one standard deviation.

Under SD conditions, the maturity R7 stage (Fehr *et al*., 1971) of the WT plants, *ft2a* mutants, *ft5a* mutants and *ft2aft5a* double mutants was 77.2 DAE, 80.4 DAE, 76.1 DAE and 97.7 DAE, respectively (Figure 3a). The WT plants produced an average of two additional nodes after floral induction and terminated the stem growth prematurely, whereas the *ft2a* mutants exhibited late flowering by about 6 days and maintained a relatively long period of vegetative growth after flowering, ultimately resulting in higher heights and the growth of an average of four additional as compared to the WT plants. In contrast, the plant height and node number of the *ft5a* mutants were not significantly different from WT plants (Figure 3b,c,d). It is highly notable that the vegetative growth of the *ft2aft5a* double mutants under SD conditions was very vigorous, and they flowered at 57.4 ± 3.5 DAE, about 31.3 days later than that of WT plants. The number of nodes per *ft2aft5a* double mutant was 20.0 ± 0.9, while that of WT plants was only 10.3 ± 0.7 (Figure 3b,c). These mutants reached a height of 235 cm, over double the height of WT plants (Figure 3b,d).

In general, the flowering position of WT plants was mainly at the leaf axil; flower stalks are located at this position. The *ft2a* mutants and *ft5a* mutants had the same flowering position as the WT plants, whereas most nodes of the *ft2aft5a* double mutants first produced many new branches instead of flowers at the leaf axil and then produced flower buds at these new branches (Figure 3e). Importantly, the branches at the leaf axils of the *ft2aft5a* double mutants produced more pods than the WT, thereby increasing the number of pods at each node (Figure 3f). Along with the increased number of nodes, the total numbers of pods and seeds of per *ft2aft5a* double mutant (pods 54.7 ± 12.6; seeds 106.3 ± 22.8) were significantly more than that of WT plants (pods 11.9 ± 3.2; seeds 27.7 ± 6.4) (Figure 3g,h).

### Expression of flowering‐related genes in the shoot apex under SD and LD conditions

To explore how *GmFT2a* and *GmFT5a* regulate flowering differently under SD and LD conditions, we focused on several downstream flowering‐related genes which have been isolated and characterized in soybean (Chi *et al*., 2011; Jia *et al*., 2015; Meng *et al*., 2007; Na *et al*., 2013; Nan *et al*., 2014; Zhong *et al*., 2012). We analysed the expression level of such genes, including *GmAP1a* (Glyma16g13070), *GmAP1b* (Glyma01g08150), *GmAP1c* (Glyma08g36380), *GmLFY2* (Glyma06g17170), *GmFULa* (Glyma06g22650), *GmFULb* (Glyma04g31847), *GmAG* (Glyma15g09500), *GmSOC1a* (Glyma18g45780) and *GmSOC1b* (Glyma09g40230), in shoot apices. *GmFT2a* and *GmFT5a* expression were significantly up‐regulated, respectively, in *GmFT2a*‐ox and *GmFT5a*‐ox plants (Figures S4 and S5). The expression levels of *GmAP1* (*a*,* b*,* c*), *GmLFY2*,* GmFULa*,* GmFULb* and *GmAG* were significantly up‐regulated in *GmFT2a*‐ox plants (Figure S4), findings consistent with the extreme early‐flowering phenotype observed for these plants under both SD and LD conditions. Under SD conditions, the expression levels of *GmAP1* (*a*,* b*,* c*) and *GmAG* were not affected, while those of *GmLFY2*,* GmFULa* and *GmFULb* were slightly but not significantly up‐regulated in *GmFT5a*‐ox plants. In contrast, under LD conditions, *GmAP1* (*a*,* b*,* c*) and *GmAG* expression were significantly up‐regulated in *GmFT5a*‐ox plants (Figure S5), which may contribute to its early‐flowering phenotype.

**Figure 4:**
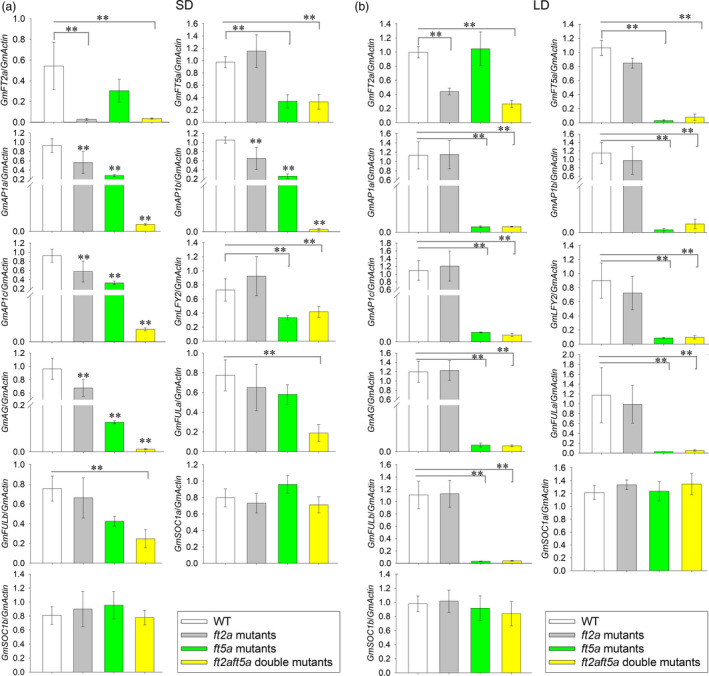
Expression analyses of *GmFT2a*,* GmFT5a* and flowering‐related genes in the shoot apices of the WT plants, *ft2a* mutants, *ft5a* mutants and *ft2aft5a* double mutants. (a) Expression analyses under SD (12 h light/12 h dark) conditions. (b) Expression analyses under LD (16 h light/8 h dark) conditions. Relative transcript levels of these genes were normalized to *GmActin*. Average and SE (standard error) values for three replications are shown in these bar plots. **P < 0.01.

As shown in Figure 4, *GmFT2a* and *GmFT5a* expression were significantly down‐regulated in *ft2a* mutants and *ft5a* mutants, respectively. They were also significantly down‐regulated in *ft2aft5a* double mutants. Under SD conditions, *GmAP1* (*a*,* b*,* c*) and *GmAG* were significantly down‐regulated in *ft2a* mutants and *ft5a* mutants, with particularly pronounced down‐regulation in the *ft2aft5a* double mutants. The expression of neither *GmFULa* nor *GmFULb* was significantly affected in the *ft2a* mutants or in the *ft5a* mutants, but they were significantly down‐regulated in the *ft2aft5a* double mutants. Viewed in combination with the flowering phenotype results mentioned above, we suggest that *GmFT2a* and *GmFT5a* may act as the primary activators of flowering, even when either of them is non‐functional. *GmFT2a* has more significant effects on floral transition than that of *GmFT5a* under SD conditions. *GmLFY2* expression was slightly down‐regulated in *ft5a* mutants and *ft2aft5a* double mutants, but not in *ft2a* mutants. Under LD conditions, *GmAP1* (*a*,* b*,* c*), *GmLFY2*,* GmAG*,* GmFULa* and *GmFULb* were not affected in the *ft2a* mutants, whereas the expression of these genes was significantly down‐regulated in *ft5a* mutants and *ft2aft5a* double mutants, providing further evidence that *GmFT5a* is a crucial gene for soybean flowering under LD conditions. We did not find any significant expression change in *GmSOC1a* or *GmSOC1b* in the aforementioned plants under SD or LD conditions (Figure 4, Figures S4 and S5).

### Haplotyping and analysis of phenotypic variation under natural field conditions in five different latitudes

**Figure 5:**
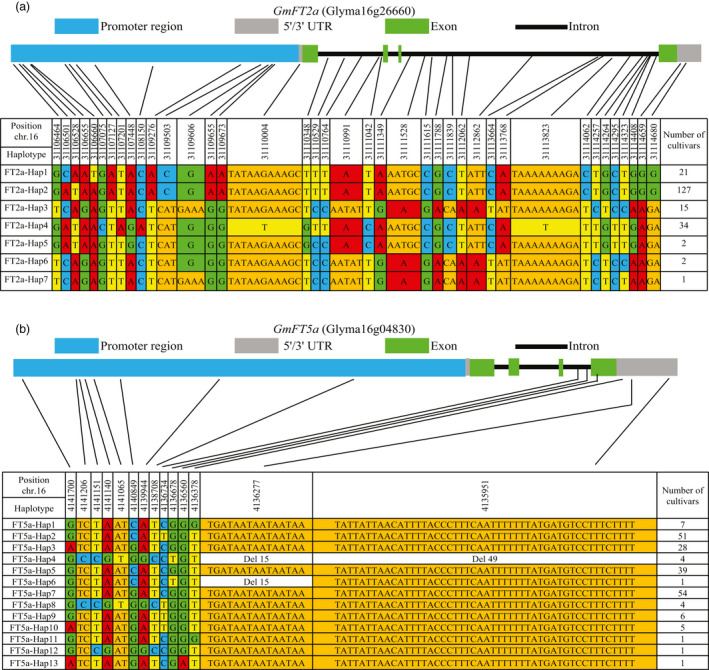
Haplotypes of *GmFT2a* and *GmFT5a*. *GmFT2a* and *GmFT5a* gene regions of 202 soybean accessions were compared with those of Williams 82. Site numbering and physical positions are also based on the reference genome sequence of Williams 82. (a) Haplotypes of *GmFT2a*. (b) Haplotypes of *GmFT5a*. Nucleotides are highlighted in different colours. The number of cultivars belong to each haplotype is listed in the right column.

We subsequently examined the nucleotide polymorphisms in the coding and non‐coding regions of the *GmFT2a* and *GmFT5a* loci in a diversity panel comprising 202 soybean accessions with varied flowering time. No frameshifts or premature stop codons were found in the coding regions of *GmFT2a* or *GmFT5a*. We also found that there was clear linkage disequilibrium for the *GmFT2a* or *GmFT5a* loci in this diversity panel (Figure S6). For *GmFT2a*, seven haplotypes were identified, and four major haplotypes (FT2a‐Hap1, FT2a‐Hap2, FT2a‐Hap3 and FT2a‐Hap4) with higher frequencies in the panel were further analysed (Figure 5a). For *GmFT5a*, thirteen haplotypes were identified, and four major haplotypes (FT5a‐Hap2, FT5a‐Hap3, FT5a‐Hap5 and FT5a‐Hap7) with higher frequencies in the panel were analysed in greater detail (Figure 5b).

The flowering time phenotypes were also recorded for plants grown at five sites in China with different latitudes: Sanya (N18°21′, E109°10′), Hunan (N27°49′, E112°56′), Beijing (N40°09′, E116°14′), Changchun (N43°49′, E125°21′) and Heihe (N50°15′, E127°27′). Consistent with its most northerly position, the soybean accessions grown at Heihe showed the longest flowering time of the five environments. Furthermore, by comparing the flowering times of the different *GmFT2a* or *GmFT5a* haplotypes (Figure S7a) and these combined haplotypes (Figure S7b), we found that haplotype FT5a‐Hap2 showed earlier flowering time in the five environments, and the phenotypic tendency becomes more pronounced with the increasing latitude. We also found that the haplotype FT5a‐Hap3 played an important role in ‘extremely’ late flowering, especially at higher latitudes (Figure S7a). The combined haplotype FT2a‐Hap2/FT5a‐Hap2 exhibited significant early flowering in all five environments. All varieties with the FT2a‐Hap3/FT5a‐Hap3 haplotypes could not flower normally when they were grown at Heihe (higher latitude) (Figure S7b). In short, we conclude that different *GmFT2a* and *GmFT5a* haplotypes have considerable effects on the diversity of flowering time in soybean at different latitudes.

### Distributions of major *GmFT2a*/*GmFT5a* haplotypes in soybean varieties from diverse geographical origins and maturity groups

Six ecological regions or ten subareas of soybean have been delineated in China on the basis of the climatic and geographical conditions, cropping systems, season sowing types and maturity group (MG) types (Wang and Gai, 2002). We analysed the geographic distribution of varieties with major *GmFT2a*/*GmFT5a* haplotypes. FT2a‐Hap1 was mostly found in the Huanghuaihai double cropping planting eco‐region (Figure S8a). FT2a‐Hap2 was mostly found in higher latitude region in the northern single cropping planting eco‐region and the Huanghuaihai double cropping planting eco‐region (Figure S8b). Accessions of FT2a‐Hap3 were present in the four eco‐regions located south of N32°18′ in China (Figure S8c). The geographic distribution of FT2a‐Hap4 was comparatively wide, but was rare in the northeast spring planting ecological subarea (Figure S8d).

**Figure 6:**
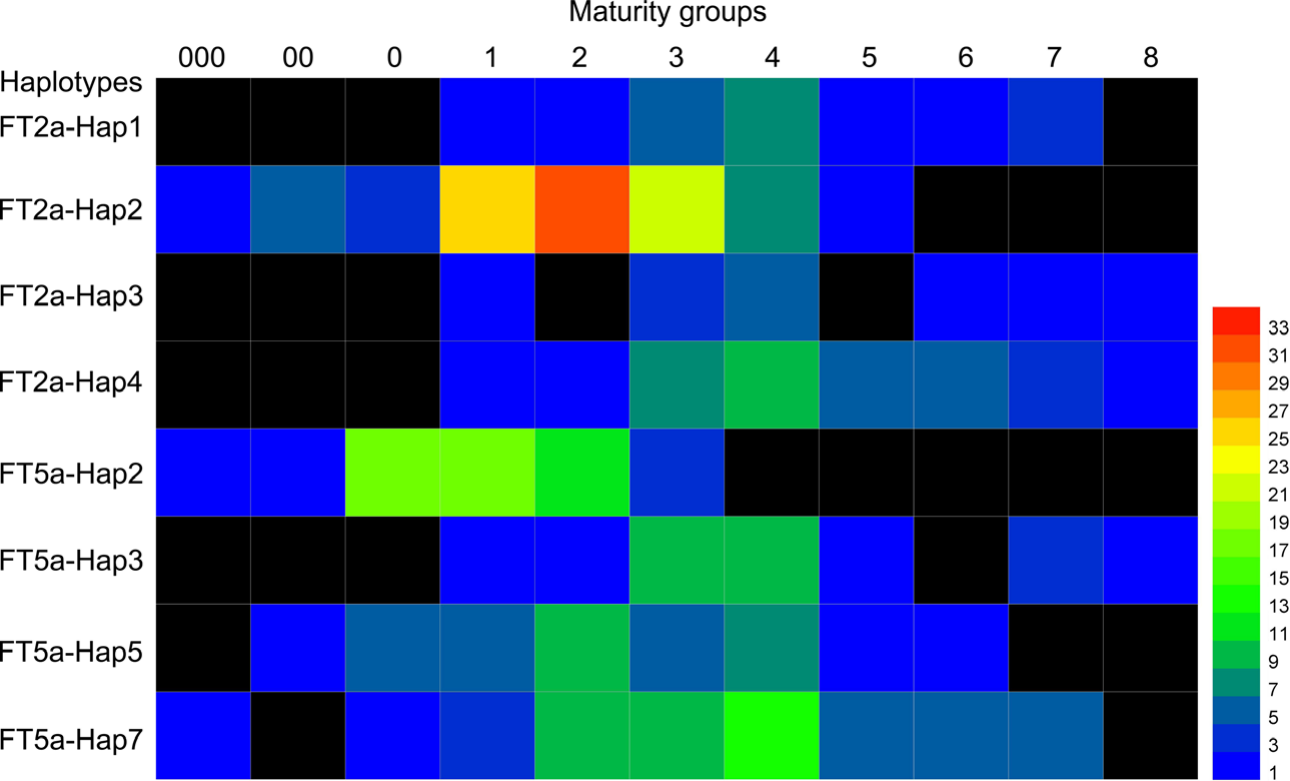
Maturity groups of the soybean accessions with major haplotypes of *GmFT2a* and *GmFT5a*. The number next to coloured bar indicates the number of soybean accessions with the corresponding haplotype for *GmFT2a* or *GmFT5a*.

Notably, FT5a‐Hap2 was only found in a higher latitude region: the northern single cropping planting eco‐region (Figure S8e). FT5a‐Hap3 was mostly found in the Huanghuaihai double cropping planting eco‐region and eco‐regions in the south of the Qinling Mountains‐Huaihe River, and it was also rare in the northeast spring planting ecological subarea (Figure S8f). We further analysed the maturity groups of accessions with major *GmFT2a*/*GmFT5a* haplotypes (Figure 6). The FT2a‐Hap1, FT2a‐Hap3, FT2a‐Hap4 and FT5a‐Hap3 genotypes were not found among the earlier maturing varieties (MG 000, MG 00 and MG 0). FT2a‐Hap2 was mainly distributed in the varieties of MG 1, MG 2 and MG 3. The distributions of FT5a‐Hap5 and FT5a‐Hap7 in various maturity groups were relatively dispersed. Most notably, FT5a‐Hap2 was only found in the earlier maturing varieties (from MG 000 to MG 3). These distributions were consistent with flowering responses to day length. We suggest that the FT5a‐Hap2 genotype may contribute to early flowering of soybean varieties at high latitudes.

## Discussion

*GmFT2a* and *GmFT5a* have been mentioned in a majority of studies that examined flowering time regulation in soybean, and they are often mentioned in pairs. There are 10 *FT* homologs in soybean (Kong *et al*., 2010). Among them, *GmFT2a* and *GmFT5a* have been identified as flowering activators and integrators (Kong *et al*., 2010; Nan *et al*., 2014; Sun *et al*., 2011), whereas *GmFT1a* (Glyma18g53680) and *GmFT4* (Glyma08g47810) are known to be function as flowering repressors (Liu *et al*., 2018; Zhai *et al*., 2014). Previous studies have suggested that *GmFT2a* and *GmFT5a* can function redundantly in their roles as promoters of flowering, even though their expression levels respond differently to photoperiodic changes (Kong *et al*., 2010; Nan *et al*., 2014). However, information about the individual effects of *GmFT2a* and *GmFT5a* in floral induction and their differential regulation of downstream flowering‐related genes under SD and LD conditions remain limited. In this study, we demonstrated that the functions of *GmFT2a* and *GmFT5a* were not redundant as previously reported. *GmFT2a* and *GmFT5a* collectively regulate flowering time in soybean. Under SD conditions, the effect of *GmFT2a* was more prominent than that of *GmFT5a*, whereas *GmFT5a* had more significant effects than *GmFT2a* under LD conditions. *GmFT5a*, not *GmFT2a*, was essential for soybean to adapt to high latitude regions.

Soybean is sensitive to changes in day length. Although this photoperiod sensitivity limits its geographical range of cultivation, it has been widely introduced to many countries in various latitudes, such as Korea, Japan and in many countries of North/Central/South America. As a short‐day plant, a crucial trait for soybean to adapt to higher latitudes is a reduced or absent inhibition of flowering by LD conditions (Liu *et al*., 2016). In this study, we found four major haplotypes of *GmFT5a*. The relationship between flowering time and *GmFT5a* haplotype suggested that FT5a‐Hap2 is a functional allele contributing to early flowering at high latitudes, whereas FT5a‐Hap3 is a weakened allele contributing to ‘extremely’ late flowering. Thus, the haplotype data may be useful for flowering time prediction or make it easier to breed for varieties with the desired flowering time under LD conditions in future studies.

Many soybean varieties from mid and high latitudes that have been grown at lower latitudes flower and mature very early, which usually results in extremely low grain yields. Introduction of long‐juvenile traits could extend the vegetative phase and improve yield under SD conditions, thereby enabling expansion of cultivation in tropical regions (Lu *et al*., 2017). Moreover, the optimization of plant architecture and higher yield are also major goals of researchers. Consider that the plant architecture of soybean, including leaves, inflorescences, and pods at each node, is known to strongly influence soybean yields. Thus, to breed high‐yielding soybean varieties, the coordination between branching (branch numbers, lengths and angles) and vertical growth (main stem‐containing nodes) is required (Pedersen and Lauer, 2004). In a previous study, transgenic soybean plants overexpressing *GmmiR156b* produced significantly increased numbers of long branches, nodes, and pods, and they exhibited an increase in 100‐seed weight, achieving substantial improvements in soybean architecture and yield per plant (Sun *et al*., 2019). In the present study, we employed the CRISPR/Cas9 system to specifically knockout the soybean gene *GmFT5a* and also produced *ft2aft5a* double mutants. Under SD conditions, the *ft2aft5a* double mutants flowered at 57.4 ± 3.5 DAE, about 31 days later than WT plants, and they maintained a vertical growth habit. As the vegetative growth stage was extended, these mutants produced significantly increased numbers of nodes. Beyond that, most of their leaf axils produced new branches and then produced many more pods in these branches, resulting in substantial increase in numbers of pods and seeds per plant (Figure 3). In view of the adaptation to SD conditions and the increased yield per plant, we speculate that the *ft2aft5a* double mutants may have enormous potential to be introduced to the tropics.

In conclusion, we dissected individual effects of *GmFT2a* and *GmFT5a* on flowering time under LD and SD conditions, these results will contribute to more accurate studies on flowering regulation and expand the regional adaptability of fine soybean varieties in the future.

## Experimental procedures

### Genotyping and QTL analysis

In this study, the 308 RIL population was genotyped using a type IIB endonucleases restriction‐site associated DNA approach (2b‐RAD) (Wang *et al*., 2012), and a linkage map (2208.16 cm) with an average distance of 0.64 cm between adjacent markers was constructed using JoinMap 4.1 (Stam, 1993). QTL analysis for flowering time was performed according to the linkage map using QTL IciMapping software v4.1, with inclusive composite interval mapping of additive functionality (ICIM‐ADD) (Li *et al*., 2008a; Meng *et al*., 2015).

### Soybean materials and growth conditions

The sequence and detailed information of the soybean genes *GmFT2a* and *GmFT5a* was searched from the Phytozome website (phytozome.jgi.doe.gov). To construct overexpression vectors of *GmFT2a* and *GmFT5a*, the total RNA from fully developed trifoliate leaves of the soybean cultivar Jack (grown under SD conditions) was isolated using a TransZol Up Plus RNA Kit (TransGen Biotech, Beijing, China) and single‐stranded cDNA wad synthesized using TransScript One‐Step gDNA Removal and cDNA Synthesis Super Mix (TransGen Biotech, Beijing, China). The CDS of *GmFT2a* (531 bp) and *GmFT5a* (519 bp) were, respectively, amplified by primers *GmFT2a*‐ox‐F/R and *GmFT5a*‐ox‐F/R (Table S2) and then inserted into the plant binary vector pTF101.1 using a pEASY‐Uni Seamless Cloning and Assembly Kit (TransGen Biotech, Beijing, China). *GmFT2a* and *GmFT5a* were driven by a 2X CaMV 35S promoter. Each construct was transformed into *Agrobacterium tumefaciens* strain EHA101 via electroporation. The variety Jack was used for transformation according to a previously published protocol (Chen *et al*., 2018). Three transgenic *GmFT2a* T3 overexpression lines (#2, #4, #7) and three *GmFT5a* T3 overexpression lines (#1, #3, #4) were used for expression analysis of flowering‐related genes. The expression levels of *GmFT2a* and *GmFT5a* were highest in the *GmFT2a* overexpression line #2 and *GmFT5a* overexpression line #3, respectively, so they were used to study the flowering phenotype. The homozygous *ft2a* mutants (1‐bp insertion at target site *GmFT2a*‐SP2, frameshift mutation) that we previously reported (Cai *et al*., 2018) were used in this study.

We employed the CRISPR/Cas9 system to specifically knockout the soybean gene *GmFT5a*. Two target sites (referred to here as *GmFT5a*‐SP1 and *GmFT5a*‐SP2) in the first exon of *GmFT5a* were selected (Figure S9a) using the web tool CRISPR‐P (Lei *et al*., 2014). Pairs of DNA oligonucleotides of the two sgRNAs were synthesized by TSINGKE (Beijing) and annealed to generate dimers, which were subsequently integrated into the CRISPR/Cas9 expression vector we previously reported (Cai *et al*., 2018). These vectors were then individually transformed into *Agrobacterium tumefaciens* strain EHA105 via electroporation. The soybean variety Jack was used for transformation according to a previously reported protocol (Chen *et al*., 2018). We totally detected 34 T1 homozygous *ft5a* mutants, and obtained two types of mutations at target site *GmFT5a*‐SP1 (Figure S9b,c) and five types of mutations at target site *GmFT5a*‐SP2 (Figure S9b,d). We then performed hybridization using T1 homozygous *ft5a* mutants (2‐bp insertion at target site *GmFT5a*‐SP2) as the male parent and *ft2a* mutants (1‐bp insertion) as the female parent to generate *ft2aft5a* double mutants.

In this study, the culture rooms under LD (16 h light/30 °C and 8 h dark/22 °C), 14H (14 h light/30 °C and 10 h dark/22 °C), 15H (15 h light/30 °C and 9 h dark/22 °C), SD (12 h light/30 °C and 12 h dark/22 °C) and SD13‐LD (transferred to LD after 13 days of SD treatment) photoperiodic conditions were used. The red‐to‐blue quantum (R:B) ratio of the light was 5.03, while the red‐to‐far‐red quantum (R:FR) ratio of the light was 3.26.

### Phenotyping and statistical analysis

The flowering time of each soybean plant was recorded as days from emergence to the R1 stage (one flower at any node), and the physiological maturity was recorded as days from emergence to the R7 stage (any pod becomes to the mature colour) (Fehr *et al*., 1971). The plant height was measured from cotyledon node to stem tip. The cotyledon node was counted as the first node. We also counted the number of pods and seeds per plant. Statistical analyses were performed using Microsoft Excel. A one‐way analysis of variance least significant difference test (LSD) was used to compare the significance of differences between controls and treatments at the 0.01 probability level. SigmaPlot 10.0 was used for drawing bar plots. These data are shown as the mean values ± one standard deviation.

### Gene expression analysis by quantitative real‐time PCR

To examine the expression of flowering‐related genes in shoot apex, the shoot apices of the WT plants, *GmFT2a*‐ox plants, *GmFT5a*‐ox plants, *ft2a* mutants, *ft5a* mutants and *ft2aft5a* double mutants under SD conditions were sampled at 13 DAE. The WT plants and *GmFT2a*‐ox plants under LD conditions were sampled at 13 DAE. The WT plants, *GmFT5a*‐ox plants, *ft2a* mutants, *ft5a* mutants and *ft2aft5a* double mutants under LD conditions were sampled at 30 DAE. Total RNA was extracted using a Quick‐RNA MicroPrep Kit (Zymo Research, Beijing, China). First‐strand cDNA was synthesized from the total RNA using TransScript One‐Step gDNA Removal and cDNA Synthesis Super Mix (TransGen Biotech, Beijing, China). For qRT‐PCR, each 10 μL reaction contained 1 μL 1:5 diluted cDNA with 0.2 μL upstream and downstream primers (10 μm), 3.6 μL ddH_2_O and 5 μL ChamQ SYBR^®^ qPCR Master Mix (Vazyme Biotech, Nanjing, China). The ABI QuantStudio^™^ 7 Flex Real‐Time PCR System was used. The PCR cycling conditions were 95 °C for 30 s, followed by 40 cycles of 95 °C for 10 s and a primer extension reaction at 60 °C for 30 s. All PCRs were run with three biological replicates each. The relative expression level was analysed using the 2^−ΔΔCt^ method with the *GmActin* (Glyma18g52780) gene as an internal control. Primers used in the expression analyses are listed in Table S2.

## Conflict of interest

The authors declare that they have no conflicts of interest.

## Author contributions

Y.C. and L.W. performed the experiments. Y.C., L.W. and L.C. wrote the manuscript. T.W. and L.L. provided the data for the loci in the 202 soybean accessions of the diversity panel. W.Y. assisted in soybean transformation. S.S. and C.W. provided soybean varieties. S.S. constructed the RIL population. S.Y. and B.J. revised the manuscript. W.H. and T.H. designed and advised on the experiments and revised the manuscript.

## Supporting information


**Figure S1** Day length of the eight environments.
**Figure S2** Flowering time under SD13‐LD (shifted to LD after 13 d of SD treatment) conditions.
**Figure S3** Flowering time of WT (wild‐type) plants, *ft2a* mutants and *ft5a* mutants under SD, 14H, 15H and LD conditions.
**Figure S4** Expression analyses of *GmFT2a* and flowering‐related genes in shoot apices of three transgenic *GmFT2a* overexpression lines #2, #4, #7.
**Figure S5** Expression analyses of *GmFT5a* and flowering‐related genes in shoot apices of three transgenic *GmFT5a* overexpression lines #1, #3, #4.
**Figure S6** Linkage disequilibrium analysis in the coding and non‐coding regions of *GmFT2a* and *GmFT5a* among 202 soybean accessions.
**Figure S7** Flowering time of the soybean accessions with major haplotypes of *GmFT2a*,* GmFT5a* and combined haplotypes of *GmFT2a*/*GmFT5a* at five different latitudes.
**Figure S8** Geographic distribution of soybean accessions with major haplotypes of *GmFT2a* and *GmFT5a*.
**Figure S9** Homozygous targeted mutagenesis of *GmFT5a* induced by CRISPR/Cas9.
**Table S1** Putative QTL for soybean flowering time in RIL families across eight environments on chromosome 16.
**Table S2** Primer sequences used in the present study.
**Appendix S1** Genome sequences of *GmFT2a* and *GmFT5a* in soybean variety HH27 and ZGDD.
