# Supplementary material for: Mutagenesis of GmFT2a and GmFT5a mediated by CRISPR/Cas9 contributes for expanding the regional adaptability of soybean
Source: Plant Biotechnol J. 2019 Jul 5;18(1):298–309. doi: 10.1111/pbi.13199 (PMC6920152; doi:10.1111/pbi.13199)
Supplement: Supplementary file 1 — Figure S1 Day length of the eight environments. Figure S2 Flowering time under SD13‐LD (shifted to LD after 13 d of SD treatment) conditions. Figure S3 Flowering time of WT (wild‐type) plants, ft2a mutants and ft5a mutants under SD, 14H, 15H and LD conditions. Figure S4 Expression analyses of GmFT2a and flowering‐related genes in shoot apices of three transgenic GmFT2a overexpression lines #2, #4, #7. Figure S5 Expression analyses of GmFT5a and flowering‐related genes in shoot apices of three transgenic GmFT5a overexpression lines #1, #3, #4. Figure S6 Linkage disequilibrium analysis in the coding and non‐coding regions of GmFT2a and GmFT5a among 202 soybean accessions. Figure S7 Flowering time of the soybean accessions with major haplotypes of GmFT2a, GmFT5a and combined haplotypes of GmFT2a/GmFT5a at five different latitudes. Figure S8 Geographic distribution of soybean accessions with major haplotypes of GmFT2a and GmFT5a. Figure S9 Homozygous targeted mutagenesis of GmFT5a induced by CRISPR/Cas9. Table S1 Putative QTL for soybean flowering time in RIL families across eight environments on chromosome 16. Table S2 Primer sequences used in the present study. Appendix S1 Genome sequences of GmFT2a and GmFT5a in soybean variety HH27 and ZGDD. [file PBI-18-298-s001.pdf]

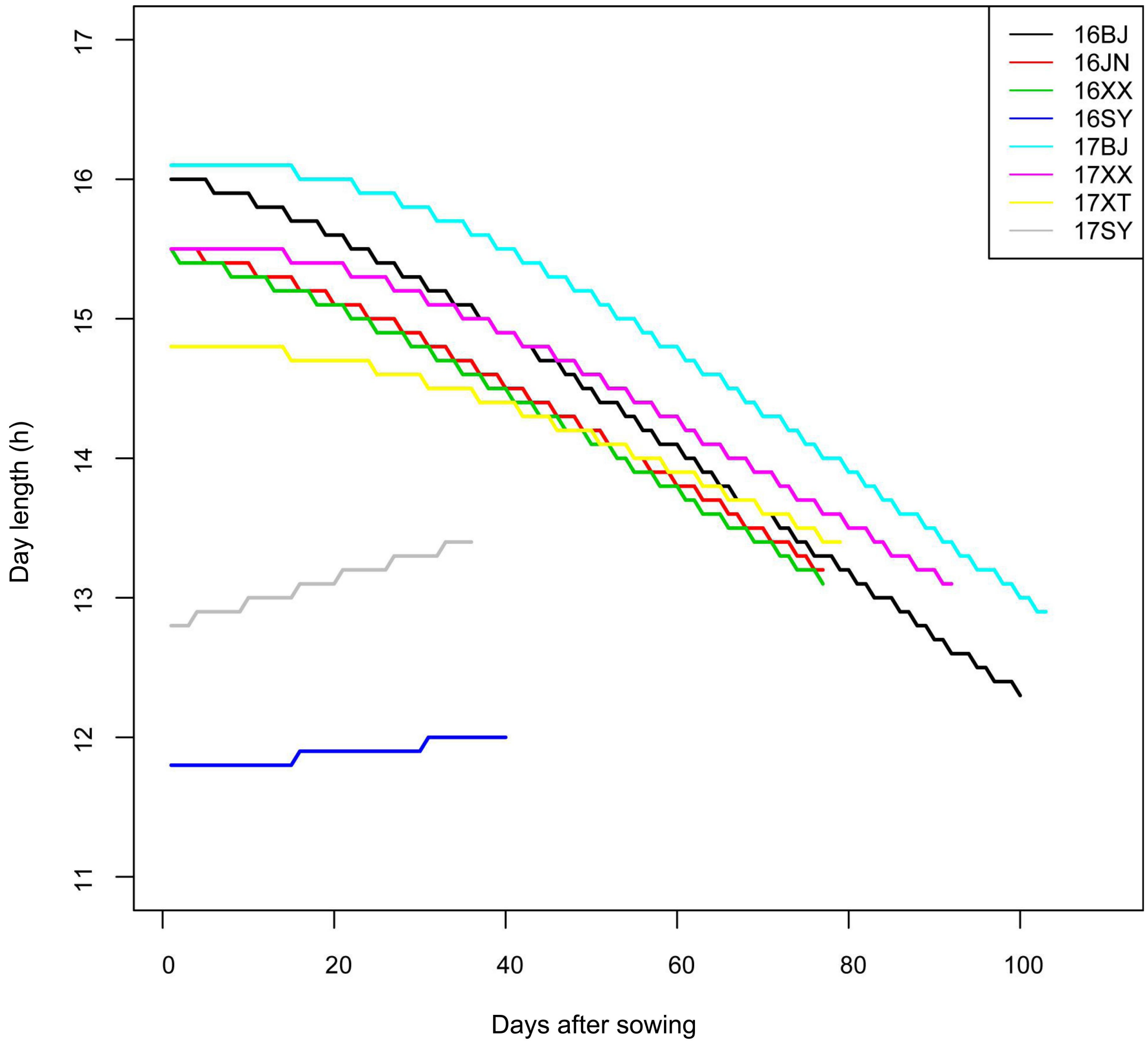

Figure S1 Day length of the eight environments. The eight environments, Beijing on July 3, 2016 and June 17, 2017, Xinxiang on July 5, 2016 and June 22, 2017, Sanya on December 19, 2016 and March 18, 2017, Jining on July 3, 2016 and Xiangtan on June 20, 2017, were named 16BJ, 17BJ, 16XX, 17XX, 16SY, 17SY, 16JN and 17XT, respectively.

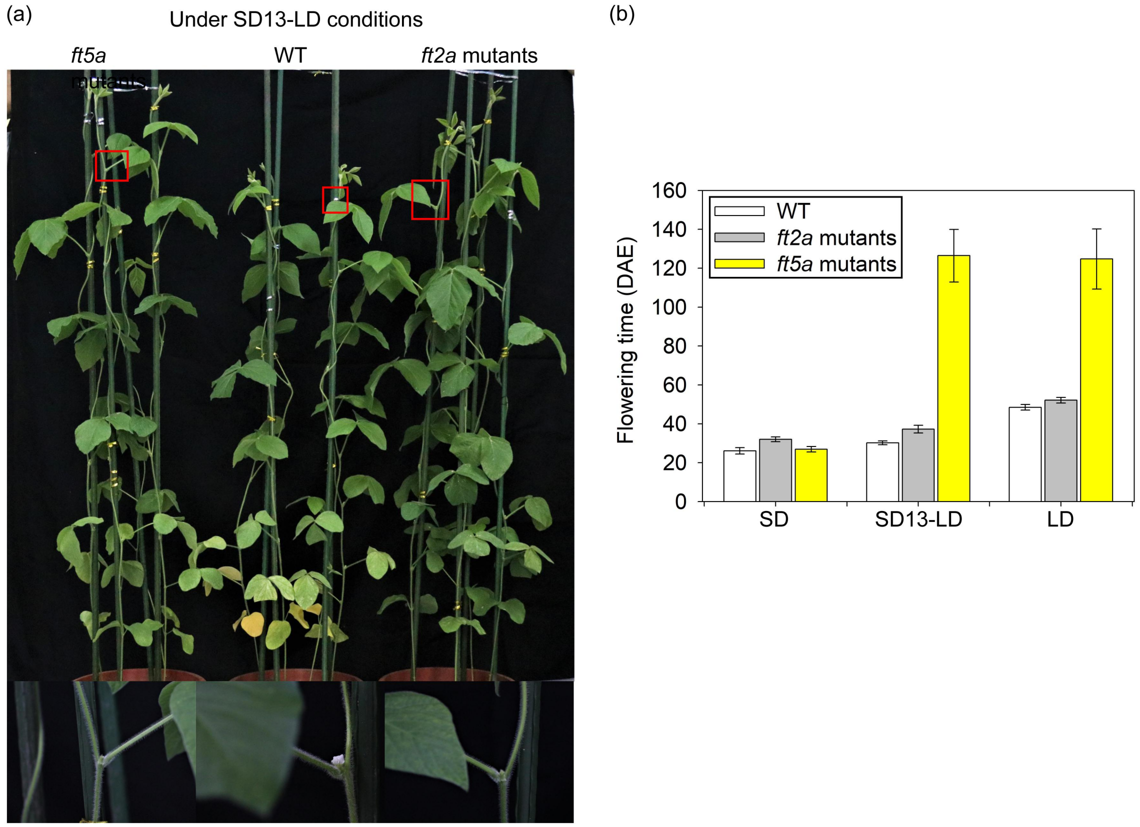

Figure S2 Flowering time under SD13-LD (shifted to LD after 13 d of SD treatment) conditions. (a) Flowering phenotype of WT (wild type) plants, *ft2a* mutants and *ft5a* mutants under SD13-LD conditions. Red box, magnified view. (b) The comparison of flowering time among WT plants, *ft2a* mutants and *ft5a* mutants under SD13-LD, SD (12 h light/12 h dark) and LD (16 h light/8 h dark) conditions. DAE, days after emergence. The flowering time values are shown as the mean ± one standard deviation.

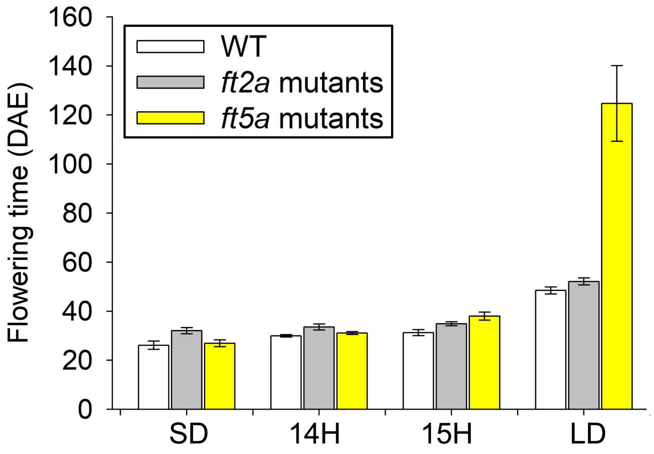

Figure S3 Flowering time of WT (wild type) plants, *ft2a* mutants and *ft5a* mutants under SD, 14H, 15H and LD conditions. SD, 12 h light/12 h dark. 14H, 14 h light/10 h dark. 15H, 15 h light/9 h dark. LD, 16 h light/8 h dark. DAE, days after emergence. The flowering time values are shown as the mean ± one standard deviation.

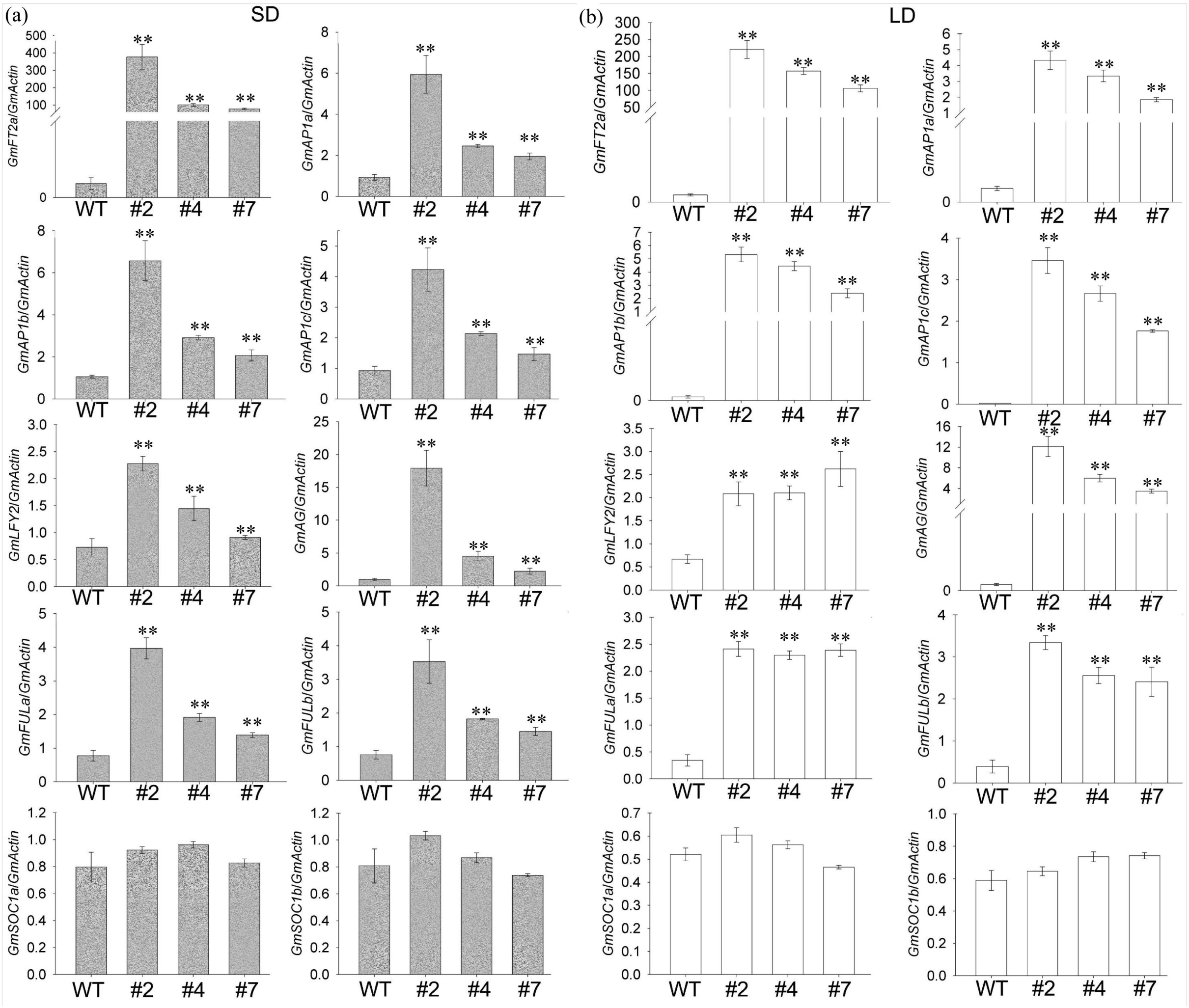

Figure S4 Expression analyses of *GmFT2a* and flowering-related genes in shoot apices of three transgenic *GmFT2a* overexpression lines #2, #4, #7. (a) Expression analyses under SD (12 h light/12 h dark) conditions. (b) Expression analyses under LD (16 h light/8 h dark) conditions. WT, wild type. Relative transcript levels of these genes were normalized to *GmActin*. Average and SE (standard error) values for three replications are shown in these histograms. **, $P < 0.01$.

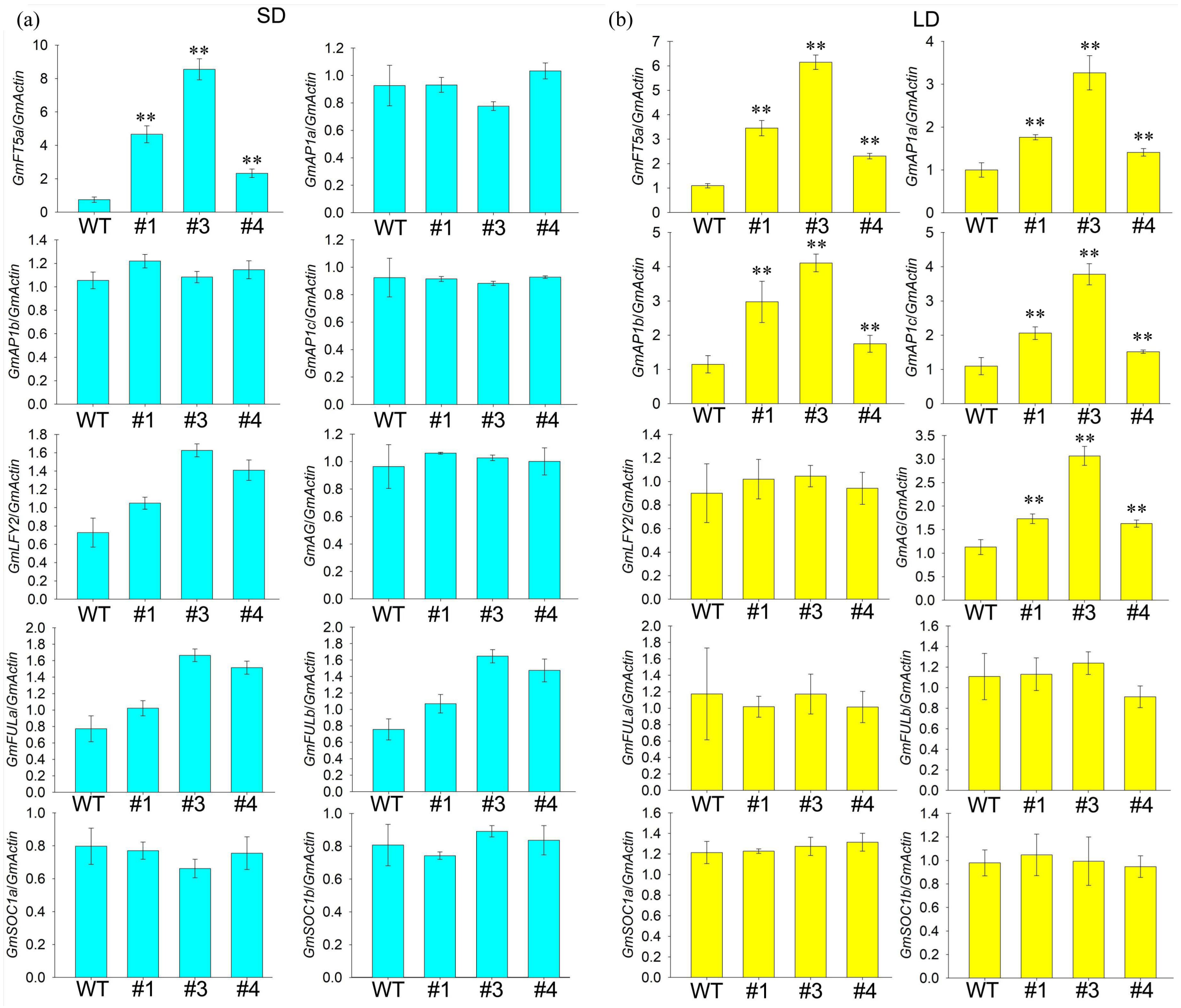

Figure S5 Expression analyses of *GmFT5a* and flowering-related genes in shoot apices of three transgenic *GmFT5a* overexpression lines #1, #3, #4. (a) Expression analyses under SD (12 h light/12 h dark) conditions. (b) Expression analyses under LD (16 h light/8 h dark) conditions. WT, wild type. Relative transcript levels of these genes were normalized to *GmActin*. Average and SE (standard error) values for three replications are shown in these histograms. **, $P < 0.01$.

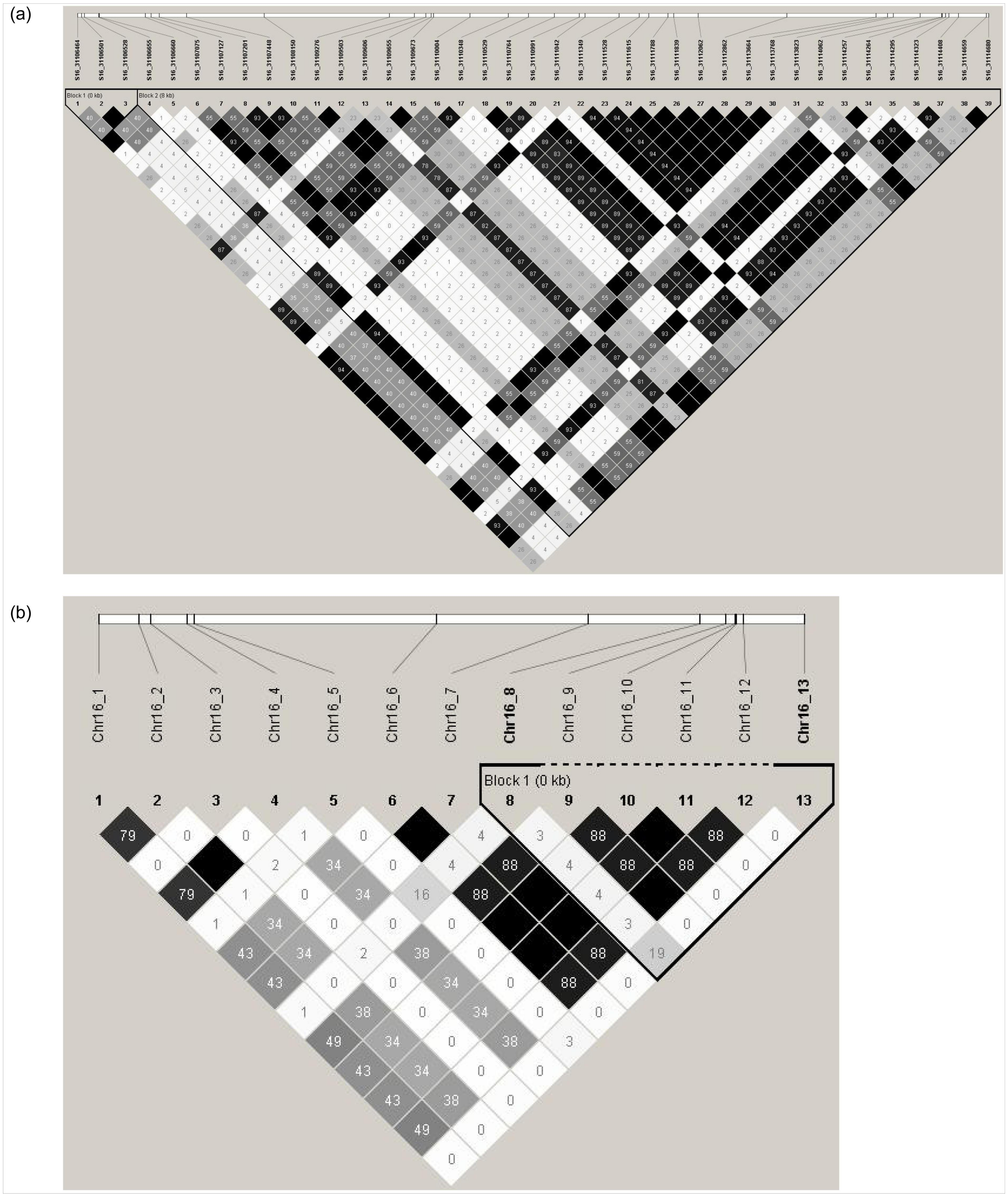

Figure S6 Linkage disequilibrium analysis in the coding and non-coding regions of *GmFT2a* and *GmFT5a* among 202 soybean accessions. (a) *GmFT2a*. (b) *GmFT5a*.

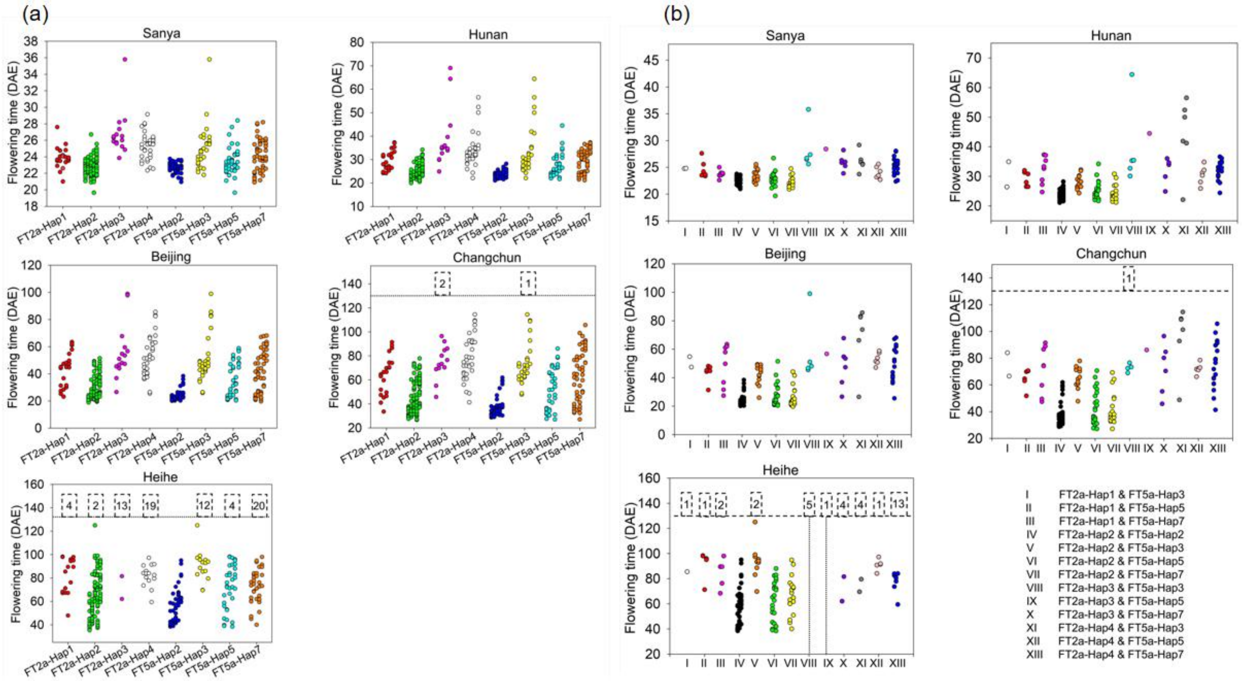

Figure S7 Flowering time of the soybean accessions with major haplotypes of *GmFT2a*, *GmFT5a* and combined haplotypes of *GmFT2a*/*GmFT5a* at five different latitudes. (a) Flowering time of the soybean accessions with major haplotypes of *GmFT2a* and *GmFT5a* at five different latitudes. (b) Flowering time of the soybean accessions with combined haplotypes of *GmFT2a*/*GmFT5a* at five different latitudes. Each dot represents a variety. DAE, days after emergence. The dotted line represents the flowering time of 130 DAE. The number within each dotted box indicates the number of varieties that did not flower after 130 DAE.

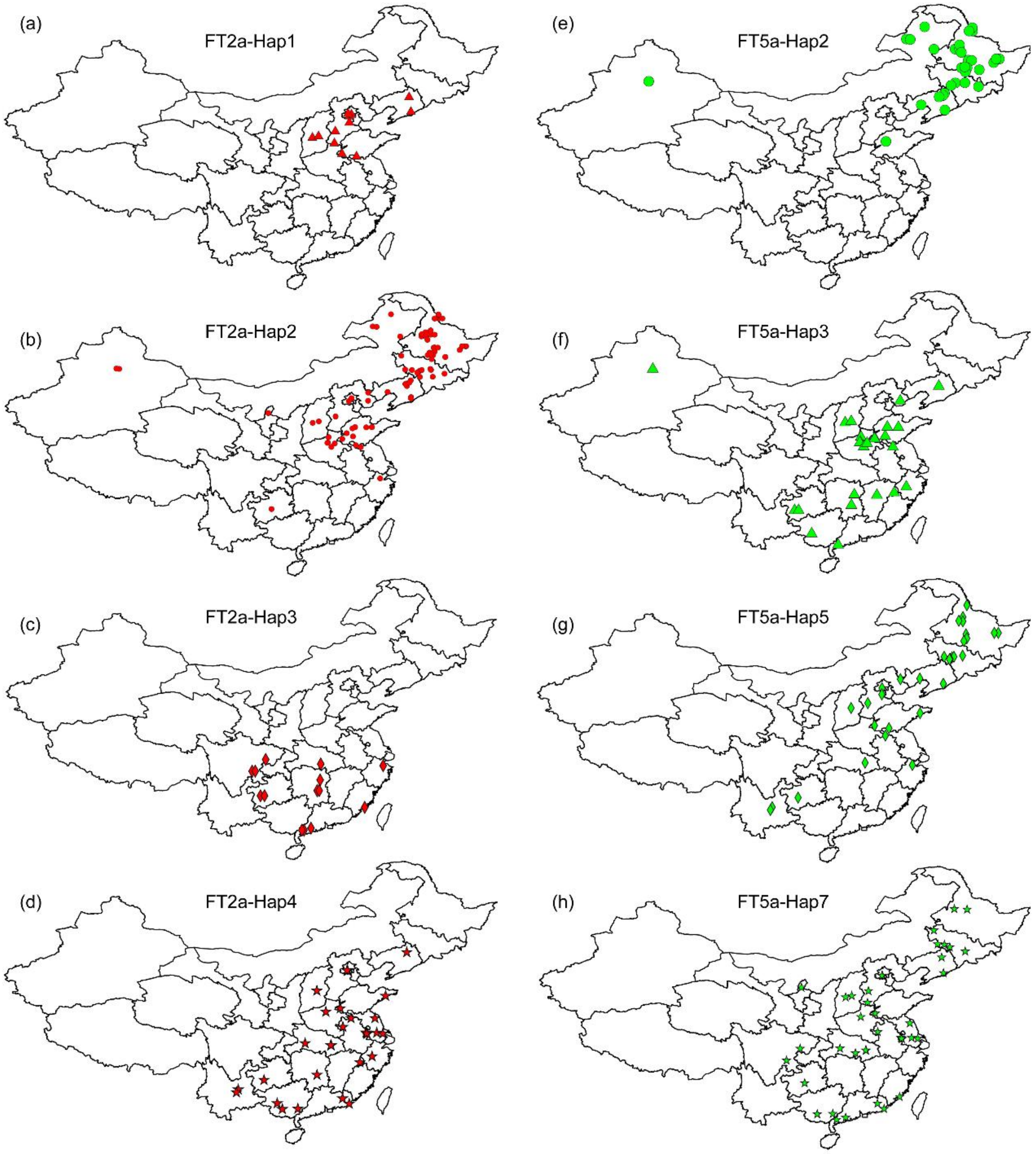

Figure S8 Geographic distribution of soybean accessions with major haplotypes of *GmFT2a* and *GmFT5a*. The map is of China. Each dot represents a variety with the corresponding haplotype for *GmFT2a* or *GmFT5a*.

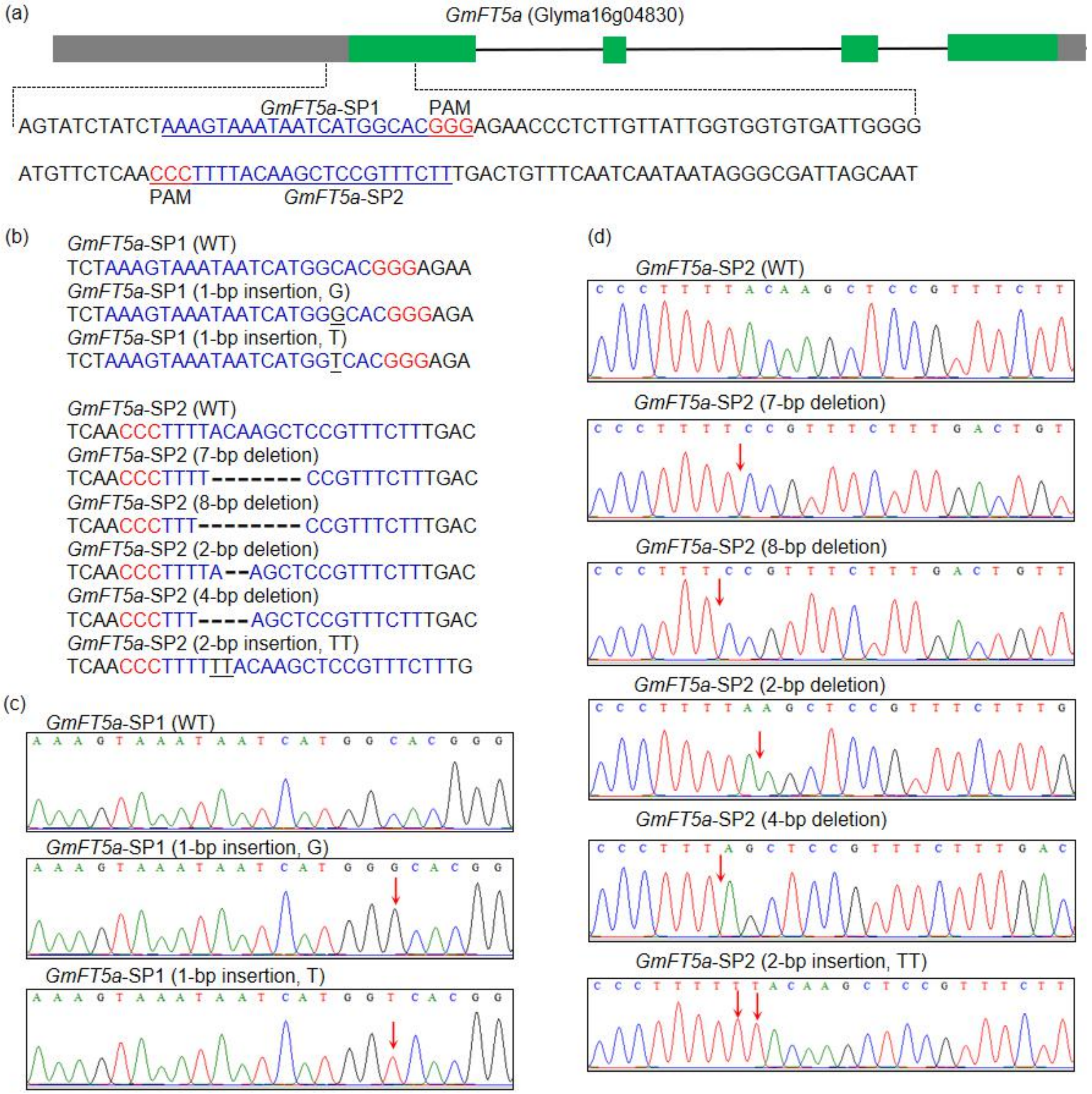

Figure S9 Homozygous targeted mutagenesis of *GmFT5a* induced by CRISPR/Cas9. (a) Gene structures of *GmFT5a* with target sites of CRISPR/Cas9. Green stripe, exon. Black line, intron. Grey stripe, UTR (untranslated regions). The underlined nucleotides indicate the target sites (named *GmFT5a*-SP1 and *GmFT5a*-SP2). PAM, protospacer adjacent motif. (b) Sequences of WT (wild type) and representative mutation types induced at *GmFT5a*-SP1 and *GmFT5a*-SP2 are presented, respectively. Underline, insertions. Dashes, deletions. (c) and (d) are sequence peaks of WT and representative mutation types at *GmFT5a*-SP1 and *GmFT5a*-SP2, respectively. The red arrowheads indicate the location of mutations.

Table S1 Putative QTL for soybean flowering time in RIL families across eight environments on chromosome 16.

QTL	Marker interval	Range (cM)	Size (Mb)	Environment	Additive effect	PVE (%)[a]	LOD
qFT16-1	Chr16-3664326-Chr16-4720071	8.05	1.06	16SY	0.65	5.04	9.47
				16XX	4.07	15.93	44.36
				16JN	4.69	19.45	46.00
				16BJ	6.52	20.07	51.04
				17SY	0.26	2.90	3.51
				17XT	3.43	12.23	25.53
				17XX	4.19	11.05	30.63
				17BJ	6.75	17.84	47.97
qFT16-2	Chr16-30470481-Chr16-33434040	21.36	2.96	16SY	1.24	18.66	23.82
				16XX	2.22	4.67	11.58
				16JN	2.32	4.74	11.48
				16BJ	2.78	3.61	8.65
				17SY	0.55	12.82	10.88
				17XT	1.76	3.20	5.97
				17XX	1.90	2.24	5.24
				17BJ	2.21	1.88	5.18

[a] Percentage of phenotypic variation explained by the corresponding QTL.

Table S2 Primer sequences used in the present study.

Primer name	Primer sequence (5'-3')	Purpose
GmFT2a-ox-F	TTGGAGAGAACACGGGGGACTCTAGAATGCCTAGTGGAAGTAGGGATCCT	To amplify the CDS of *GmFT2a*
GmFT2a-ox-R	TCCTCGCCCTTGCTCACCATTCTAGAGTATAACCTCCTTCCACCAGAACC	
GmFT5a-ox-F	GAGAACACGGGGGACTCTAGAATGGCACGGGAGAACCCTCTTG	To amplify the CDS of *GmFT5a*
GmFT5a-ox-R	CCCTTGCTCACCATTCTAGAATATCTCCTTCCACCGCAACCACGC	
q*GmAP1a*-156-F	TGAACATGGGTGGCAATTAC	Expression analysis of *GmAP1a*
q*GmAP1a*-156-R	TGTCAAATGCCATACCAAAG	
q*GmAP1b*-108-F	TGGGAGCAGCCAAACTACAG	Expression analysis of *GmAP1b*
q*GmAP1b*-108-R	TGGTGCAGCTTCCTGATTGT	
q*GmAP1c*-184-F	GAAAGAAAAGGTTGCAGCTTC	Expression analysis of *GmAP1c*
q*GmAP1c*-184-R	GCATCCAAGGTGACAGGAAT	
q*GmSOC1a*-152-F	CGAGTTGCTTTTTTTCCCTAG	Expression analysis of *GmSOC1a*
q*GmSOC1a*-152-R	TGAGTCTTTCCTCTCACCAT	
q*GmSOC1b*-147-F	AAGAAGCCCAACTGCAATGT	Expression analysis of *GmSOC1b*
q*GmSOC1b*-147-R	GGGCTTCAGAAATGAGGAAAGG	
q*GmLFY2*-162-F	TGACGAAGGAAACATTAACACTGG	Expression analysis of *GmLFY2*
q*GmLFY2*-162-R	GCCTGAACCTGCATCAAGAA	
q*GmAG*-254-F	CAACAACCAGCTTCTTCGAGC	Expression analysis of *GmAG*
q*GmAG*-254-R	AGAAGGCAAACTACAAGGTAGC	
q*GmFULa*-217-F	CTCCCACAACAACACTAGCTC	Expression analysis of *GmFULa*
q*GmFULa*-217-R	CCTACAAGACAATTCCAACACGA	
q*GmFULb*-207-F	AGTGCCCACAAAATGGAACT	Expression analysis of *GmFULb*
q*GmFULb*-207-R	TGCTTTCATGTGTACTGGGC	
q*GmFT2a*-184-F	ATGCACCTAGCCCAAGTGAC	Expression analysis of *GmFT2a*
q*GmFT2a*-184-R	TACACGGTCTCCCTACCCAG	
q*GmFT5a*-171-F	CACGGGAGAACCCTCTTGTTAT	Expression analysis of *GmFT5a*
q*GmFT5a*-171-R	GGTCTTCACCACCAACAGTAACC	
q*GmActin*-F	CGGTGGTTCTATCTTGGCATC	Expression analysis of *GmActin*
q*GmActin*-R	GTCTTTCGCTTCAATAACCCTA	

Appendix S1 Genome sequences of *GmFT2a* and *GmFT5a* in soybean variety HH27 and ZGDD.

Genome sequence of *GmFT2a* in HH27   Location: Chr16: 31109999-31114963

GAGTATATAAGAAAGCATAAGCCAAATTTTGAGTAAACTAGTGTGCACACTATCCCATGCCTAGTGGAAGTAGGGATCCTCTCGTTG
TTGGGGGAGTAATTGGGGATGTATTGGATCCTTTTGAATATTCTATTCCTATGAGGGTTACCTACAATAACAGAGATGTCAGCAATGG
ATGTGAATTCAAACCCTCACAAGTTGTCAACCAACCAAGGGTAAATATCGGTGGTGATGACCTCAGGAACTTCTATACTTTGGTAAC
TCATTAATTTTGTCCAAGTACTCTTTTTGTTGTTCATATTTATAGTGATTTTAAATTGTAGTAGTAATTTTTTATACCAAGCTAGAATAAT
TTTTGTGAGTTTTTCTATTGAAAATATAAAACTTTTTTTAATGGAGAAACTCTCAAAAACTCTTTTAACTTTGTATTCAAACAATCTCA
TAATTATTAGTAAAAAATTTAAATATGATTTTAGTTTCATTAAAATGAGCAAATTTTATTTTTAATATTTGTAAAAAGAAATTGATTTTC
TTTTTCTTTTTCTAAGAATATGACGTTATTGTTTTTGACCATCCATACTACTATTATATGATATAGCATCCATTAATTGTTTGTGTTTAAG
GGAAGGTCAAGTACCCTCTCAAGTGGACATGTACATACATGTGAGGACACCTTTATAATATTTTCTACATCCCAAAATAAATAGTATT
ATTTATCTATCTCTTTTTAATGTATTATTTATGTATCTTAAAGTAAGTTAGGGATTGAAAATATTGATATCTTATTTTAAAGGAATGAAAA
TCAATCATTTTAGAAAAATTAAAACTAAAAATTTGCTCATTGCAGAAAGTAAAGTAAAGAAAACCCCACCACTCATTTTATAGGACT
TAAAGTCATACTTAAACCTTACTAAAATTTCAAGAATTGAAATACGGATACAAGCAGTTAATGCAATATATTCTTAGTGCAATCGAGG
ATCATTAGATGTTACAATATAGTAGTAGTTTCTAGCTTAATCCAACTTGATTTCATTTCATTTTATTAACAGATTGCGGTTGATCCCGAT
GCACCTAGCCCAAGTGACCCCAATTTGAGAGAATACCTCCATTGGTGAGCCACAAATTTTTGTTTAAACACGCCATGAATGCAAAC
ATATATATGAACTGAACTATACATTTATGAGTTTTTCTAGCTTAATGTTGTAATATTATACCCCTATCTTCTCTCAATGTGTCAGGTTGGT
GACTGATATCCCAGCAACAACAGGGGCTAGTTTCGGTACGTATATGATGTTTTTCCATTTTTAAAACCTATAATTTTCTGTGCTTAAGT
TAAACCTATTCAAACCATTATTTTTAATTTAAAATTAGTGAGTTTTATTTATGTGCATATACTGATAAAAAAAATTAGTTCACATATTGT
ATTTTTATGTATGAAGTCTATTACTGGCTTGGTCTGAACAAGTAAATAATCATATATCAAAACAAGAAAAAATATTTTAAATTAAAGTA
TAATTGAACCAAGTAAAATGCATGCTATTAGTTACCAAACTAATAAGCACAAAAAATAAAATAAAAACTGATAGAAACATGATTGAT
TACTTATTCAAATTCACCCTCGCTTGTATTCTGTGTCAAACGCACAATGTTTGCTTGCTAGCTGTTTAATTTCTTTAACAAATGGACA
CATATGCTAAGTGTATTAGTGATTTATGATTTCTAATTATTTAAATTGATAGGAACTCAGCTTTTCAAAAACATTATTATTAATTATCTTA
ACTGACGAAGGTATTAGAAATTTTATTGAACACTGATTCCTAAACTCGTAAGTTTTGGAATCAAGTGTGTTATGTGATAATAAGAAGA
TCCCCATTAATAATTATTTTACTGAGTATATATAGTGGAAGGGATTTAAATTATGGATATTCTACAAGGTTCCATCAACTTTATCTTAAA
AGTTTATCTTTTGTCCTTTGGAGCAGTACATCTCCTTTCCAACAAATGAATGATGTTCGAGTTTATCAAAATATAAATGTGGTCCAAA
ATATTTTAAAATAATTTTTATTTTTTTGATATAATAAATATTCTTTTTTATTGAGTACCTTTTTTAATTTATATCTATAATTGTTAGAATTAT
TATTTTTATTTAATTATATTAGAGAAAAATGATTTAGTTAATTAAGAGTTAAATCTCCAGTAATACACCCCCCCCCCCCCCCCCAAAA
AAAAAAGTAGCTTAATTTTAATTCATTGGCTAACACAATGTGAAATACTAATGTTATGTTGAAACGGTGCATGCATGATGTATCAAAG
CATGCGTTATGCATGGATGTAGGAATGCTAAATGTGGACTGTGTTTTCCATTGACTAGTAATTTGAGACTAATCCTATGATTATGGTCA
GGCTTAGTGACTAATTAAAAAGACTCTCAACAAGTACCTTCTTCAAGGTTCGAATTTTGTTTTATTTTTAATTTTTATTGCCAGAGAA
TCCAGCTTACAATCACGCCAACCTTTAAAATGAAGAACAGATAGAGATAAGTATATGATACTAGGGCATCATATTGTAAAGTACGATC
AGCAAACCTGATGATGACCAACATACAATCCCAATTAAAATCGGAGATTATTATGATAGATATTTAAGTTGTTTCTTTGTTTTTTTTTT
TATAAAAAAAATCTTTATACAAAACCAAATATGCATATATATATATATATATATATATATATATATATATATATATATATATATATATATAT
ATATACAGTTAGTTATACATGTGTATAAATACACACAAACATACATTTATTTTTATATATACATACAATATATAGCAGATACTCAATATTG
AAAATTAATTTTTGTTTTGATGTTTCAAAAACGTTTTTATTGGTTTTAATCTTCATATACAAATATTATTTTTTTAAATGCTTCTGTCATT
ACTTTGTGATAATATATGACATGCATCTATATATGTTACATCAACAACCATCCTTAAATAACATGTAATGTAACATATATAGTCATCGAT
GCATATTACACATTATCATGAGTGAAGATACAGGTCATTTAAAAAAAAAATCAAATTTATGAGGATTAAAACTGACAAAACAAAAATT
GATAGACATAGGTTACATTAATTAAGCCATATATATTTACAAGAATTAAGAATATATATGTTTTACCTGATTATTAAGATTCAAAATATGC
AACCAAATTAGCCAAATTATGAACCATATAAGCATTGAAGTCTTGTTTAATTTCTCTCTTCCATCAGAACTCAAGTAAGAATATATGT
AGCCATCCGAATTCTTAAAGTGCATGCATGATCATGTGCAAAAAGATGAGAAGATTCATTCAGTTCAAGAAAAAAGACACAAAGC
AACTACTGTGACTAAAGGAGAATATTCCTATTGAATAAACTACCTTTTGTTGGCAAGATAGCTCTAAAACCTTCATAGGATTTACAGA

GTTATAGAACATCATAGAAAAAGCTTCATAACTTGTTGAAGCAAATTTAAATAAGGGGAGTTAAAAGAATAAGAATTAGATTTGTTT
AAACTCCATTTCCAAACATCTTATTGACCAAAACTCAGCATCATATATAATTCAATAAATTCATCACCTTGACAAAATGTGTTCTTTCT
AAGTGAAAAAGAACCCTCCTCCATTTAAACTTTCACTTCAACCATACCCCATCAACCTACTCACCAAGGTAAGCAATCTTAACATCC
TTCTCATCTGAAATAAGAAAAATTCCTCTGATTTTTTTAAAGAATTTTGAGATCCATCATCTCTAGTGAACCCCCAATCTTTCCATAA
AAAAAGAAAAAAAAGAAAAATATTCTGCTTCCATCATCTATCTGCTTTTTAAAACCAGAGCTTACCAGTATGTATGAGTGTTTCTGG
CTTTCTGCCCTTTGCAGTACGAAATGCTAAGATATTTCCACTGAATTGAAATTACGTGTTCCCATTTCCTATTTAATTTCCTTAGTTTC
TTATCTCTTCAGTTAGAGGATGACACGTACTTAATTAAAATCAACTCTATCTTCTGATTGCTATTATATTTTTGAAGTATTATTTTTTAAT
TTGGAAGAGAGGGAAAAGGACACAAGATTAGGTGATTTGGCTCCTTTCTAAAACGTTAGGACAGCAGTAGTAGGACCATACATGT
CTGAACATTATTAATACAATACCTTATCTAAGAAGAAATATAGTACTATTTTATGTTTGACAATCAAACTGAAATATATTATCATTGCAT
GTATATAAATCATAAGAGTTTAATGAGCGTCAAAGTAGATCAAGTAGAAATCACATATATATAACTTTTAATATAATTATTATAAAAATT
AATAAATTTATTGATCATATAAAATTATTTTATATTGTGGGTATTGTAACGATAAATATGCATTATTTGCAAAGGCCATGAGGTTGTAAC
ATATGAAAGTCCAAGACCAATGATGGGGATTCATCGTTTGGTGTTTGTGTTATTTCGTCAACTGGGTAGGGAGACCGTGTATGCACC
AGGATGGCGCCAGAATTTCAACACTAAAGAATTTGCTGAACTTTACAACCTTGGATTGCCAGTTGCTGCTGTCTATTTCAACATTCA
GAGGGAATCTGGTTCTGGTGGAAGGAGGTTATACTAAGAAAAAGTACTTTATATTATTGAAAAAATAAAGTAGTATAAGCTTCGTTG
AGGGTTTCAGAAATATTAATTGGCAATCTCCCACACTCTTTAGTAGTAAATGAGTGTTTTTCAACTTAATTAAACTGAGCATACAGTG
AAATAAATTGCTAGCTCAGTTGGTAGCAGCAAGTACTCTGCATATACACATAAATGAAACTGAAGCATCTAGGTTCATTTTTCTTATT
TGTATTATCAGTTGAAGAATGTTAAAGATATCTGATATACGTAAAGTGGAAAATATAACTCGAGCATAAGTTAAAGTGA

Genome sequence of *GmFT2a* in ZGDD   Location: Chr16: 31109999-31114963

GAGTATATAAGAAAGCATAAGCCAAATTTTGAGTAAACTAGTGTGCACACTATCCCATGCCTAGTGGAAGTAGGGATCCTCTCGTTG
TTGGGGGAGTAATTGGGGATGTATTGGATCCTTTTGAATATTCTATTCCTATGAGGGTTACCTACAATAACAGAGATGTCAGCAATGG
ATGTGAATTCAAACCCTCACAAGTTGTCAACCAACCAAGGGTAAATATCGGTGGTGATGACCTCAGGAACTTCTATACTTTGGTAAC
TCATTAATTTTGTCCAAGTACTCTTTTTGTTGTTCATATTTATAGTGATTTTAAATTGTAGTAGTAATTTTTTATACCAAGCTAGAATAAT
TTTTGTGAGTTTTTCTATTGAAAATATAAAACTTTTTTTAATGGAGAAACTCTCAAAAACTCTTTTAACTTTGTATTCAAACAATCTCA
TAATTATTAGTAAAAAAATTTAAATATGATTTTAGTTTCATTAAAATGAGCAAATTTTATTTTTAATATTTGTAAAAAGAAATTGATTTCC
TTTTTCTTTTTCTAAGAATATGACGTTATTGTTTTTGACCATCCATACTACTATTATATGATATAGCATCCATTAATTGTTTGTGTTTAAG
GGAAGGTCAAGTACCCTCTCAAGTGGACATGTACATACATGTGAGGACACCTTTATAATATTTTCTACATCCCAAAATAAATAGTATT
ATTTATCTATCTCTTTTTAATGTATTATTTATGTATCTTAAAGTAAGTTAGGGACTGAAAATATTGATATCTTATTTTAAAGGAATGAAAA
TCAATCATTTTAGAAAAATTAAAACTAAAAATTTGCTCATTGCAGAAAGTAAAGTAAAGAAAACCCCACCACTCATTTTATAGGACT
TAAAGTCATACTTAAACCTTACTAAAATTTCAAGAATTGAAATACGGATACAAGCAGTTAATGCAATATATTCTTAGTGCAATCGAGG
ATCATTAGATGTTACAATATATATAGTAGTAGTTTCTAGCTTAATCCAACTTGATTTCATTACATTTTATTAACAGATTGCGGTTGATCC
CGATGCACCTAGCCCAAGTGACCCCAATTTGAGAGAATACCTCCATTGGTGAGCCACAAATTTTTGTTTAAACACGCCATGAATGC
AAACATATATATGAACTGAACTATACATTTATGAGTTTTTCTAGCTTAATGTTGTAATATTATACCCCTATCTTCTCTCAATGTGTCAGG
TTGGTGACTGATATCCCAGCAACAACAGGGGCTAGTTTCGGTACGTATATGATGTTTTTCCATTTTTAAAACCTATAATTTTCTGTGCT
TAAAGTTAAACCTATTCAAACCGTTATTTTTAATTTAAAATTAGTGAGTTTTATTTATGTGCATATACTGATAAAAAAAAATTAGTTCAC
ATATTGTATTTTTATGTATGAAGTCTATTACTGGCTTGGTCTGAACAAGTAAATAATCATATATCAAAACAAGAAAAAAATATTTTAAATT
AAAGTATAACTGAACCAAGTAAAATGCTATTAGTTACCAAACTAATAAGCACAAAAAAATAAAATAAAAACTGATAGAAACATGATT
GATTACTTATTCAAATTCAGCCTCGCTTGTATTCTGTGTCAAACGCACAATGTTTGCTTGCTAGCTGTTTAATTTCTTTAACAAATGGA
CACATATGCTAAGTGTATTAGTGATTTATGATTTCTAATTATTTAAATTGATAGGAACTCAGCTTTTCAAAAACATTATTATTAATTATCT
TAACTGACGAAGATATTAGAAATTTTATTGAACACTGATTCCTAAACTCGTAAGTTTTGGAATCAAAGTGTGTTATGTGATAATAAGA
AGATCCCCATTAATAATTATTTTACTGAGTATATATAGTGGAAGGGATTTAAATTATGGATATTCTACAAGGTTCCATCAACTTTATCTT

AAAAGTTTATCTTTTGTCCTTTGGAGCAGTACATCTCCTTTCCAACAAATGAATGATGTTCGAGTTTATCAAAATATAAATGTGGTCC
AAAATATTTTAAAATAATTTTAATTTTTTTGATATAATAAATATTCTTTTTTATTGAGTACCTTTTTTAATTTATATCTATAATTGTTAGAA
TTATTATTTTTATTTAATTATATTAGAGAAAAATGATTTAGTTAATTAAGAGTTAAATCTCCAGTAATACACCCCCCCCCCCCCCCCCCA
AAAAAAAAAGTAGCTTAATTTTAATTCATTGGCTAACACAATGTGAAATACTAATGTTATGTTGAAACGGTGCATGCATGATGTATCA
AAGCATGCGTTATGCATGGATGTAGGAATGCTAAATGTGGACTGTGTTTTCCATTGACTAGTAATTTGAGACTAATCCTATGATTATG
GTCAGGCTTAGTGACTAATTAAAAAGACTCTCAACAAGTACCTTCTTCAAGGTTCGAATTTTGTTTTATTTTTAATTTTTATTGCCAG
AGAATCCAGCTTACAATCACGCCAACCTTTAAAATGAAGAACAGATAGAGATAAGTATATGATACTAGGGCATCATATTGTAAAGTA
CGATCAGCAAACCTGATGATGACCAACATACAATCCCAATTAAAATCGGAGATTATTATGATAGATATTTAAGTTGTTTCTTTGTTTTT
TTTTATAAAAAAAATCTTTATACAAAACCAAATATGCATATATATATATATATATATATATATATATATATATATATATATATATATATATATA
TATATATACAGTTAGTTATACATGTGTATAAATACACACAAACATACATTTATTTTTATATATACATACAATATATAGCAGATACTCAATAT
TGAAAATTAATTTGTTTTGATGTTTCAAAAACGTTTTTATTGGTTTTAATCTTCATATACAAATATTATTTTTTTAAATGCTTCTGTCATT
ACTTTGTGATAATATATGACATGCATCTATATATGTTACATCAACAACCATCCTTAAATAACATGTAATGTAACATATATAGTCATCGAT
GCATATTACACATTATCATGAGTGAAGATACAGGTCATTTAAAAAAAAAAATCAAATTTATGAGGATTAAAACTGACAAAACAAAAAT
TGATAGACATAGGTTACATTAATTAAGCCATATATATTTACAAGAATTAAGAATATATATGTTTTACCTGATTATTAAGATTCAAAATATG
CAACCAAATTAGCCAAATTATGAACCATAAAAGCATTCAAGTCTTGTTTAATTTCTCTCTTCCATCAGAACTCAAGTAAGAATATATG
TAGCCATCCGAATTCTTAAAGTGCATGCATGATCATGTGCAAAAAGATGAGAAGATTCATTCAGTTCAAGAAAAAAGACACAAAGC
AACTACTGTGACTAAAGGAGAATATTCCTATTGAATAAACTACCTTTTGTTGGCAAGATAGCTCTAAAACCTTCATAGGATTTACAGA
GTTATAGAACATCATAGAAAAAGCTTCATAACTTGTTGAAGCAAATTTAAATAAGGGGAGTTAAAAGAATAAGAATTAGATTTGTTT
AAACTCCATTTCCAAACATCTTATTGACCAAAACTCAGCATCATATATAATTCAATAAATTCATCACCTTGACAAAATGTGTTCTTTCT
AAGTGAAAAAGAATCCTCCTCCATTTAAACTTTCACTTCAACCATACCCCATCAACCTACTCACCAAGGTAAGCAATCTTAACATCC
TTCTCATCTGAAATAAGAAAAATTCCTCTGATTTTTTTTAAAGAATTTTGAGATCCATCATCTCTAGTGAACCCCCAATCTTTCAATAA
AAAAAGAAAAAAAAGAAAAATATTCTGCTTCCATCATCTATCTGCTTTTTAAAACCAGAGCTTACCAGTATGTATGAGTGTTTCTGG
CTTTCTGCCCTTTGCAGTACGAAATGCTAAGATATTTCCACTGAATTGAAATTACGTGTTCCCATTTCCTATTTAATTTCCTTAGTTTC
TTATCTCTTCAGTTAGAGGATGACACGTACTTAATTAAAATCAACTCTATCTTCTGATTGTTATTATATTTTTGAAGTATTATTTTTTAAT
TTGGAAGAGAGGGAAAAGGACACAAGATTAGGTGATTTGGCTCCTTTCTAAAACGTTAGGACAGCAGTAGTAGGACCATACATGT
CTGAACATTATTAATACAATACCTTATCTAAGAAGAAATATAGTACTATTTTATGTTTGACAATCAAACTGAAATATATCATCATTTCAT
GGATATAAATCATAAGAGTTTAATGAGCGTCAAAGTAGATCAAGTAGAAATCACACATATATAACTTTTAATATAATTATTATAAAAAT
TAATAAATTTATTGATCATATAAAATTATTTTATATTGTGGGTATTGTAACAATAAATATGCATTATTTGCAAAGGCCATGAGGTTGTAA
CATATGAAAGTCCAAGACCAATGATGGGGATTCATCGTTTGGTGTTTGTGTTATTTCGTCAACTGGGTAGGGAGACCGTGTATGCAC
CAGGATGGCGCCAGAATTTCAACACTAAAGAATTTGCTGAACTTTACAACCTTGGATTGCCAGTTGCTGCTGTCTATTTCAACATTC
AGAGGGAATCTGGTTCTGGTGGAAGGAGGTTATACTAAAAAAAAAGTACTTTATATTATTGAAAAAAATAAAGTAGTATAAGCTTCGT
TGAGGGTTTCAGAAATATTAATTGGCAATCTCCCACACTCTTTAGTAGTAAATGAGTGTTTTTCAACTTAATTAAACTGAGCATACAG
TGAAATAAATTGCTAGCTCAGTTGGTAGCAGCAAGTACTCTGCATATACACATAAATGAAACTGAAGCATCTAGGTTCATTTTTCTTA
TTTGTATTATCAGTTGAAGAATGTTAAAGATATCTGATATACGTAAATTGGAAAATATAACTCGAGCATAAGTTAAAGTGA

Genome sequence of *GmFT5a* in HH27   Location: Chr16: 4135885-4137742

CCAAACACAAACATCATAGTATATAGTATCTATCTAAAGTAAATAATCATGGCACGGGAGAACCCTCTTGTTATTGGTGGTGTGATTG
GGGATGTTCTCAACCCTTTTACAAGCTCCGTTTCTTTGACTGTTTCAATCAATAATAGGGCGATTAGCAATGGCTTGGAACTCAGGC
CCTCTCAAGTTGTTAATCGCCCTAGGGTTACTGTTGGTGGTGAAGACCTAAGGACCTTCTACACTCTGGTAAGTAATTAAGTAGTATA
TATAGTGGAAATTAATCAATTGCTAGCTAGCGAGTACAAATTAGTCTTAATAAATAAGTTATTAACAGCTAGTATGCATGCTATATATAT
AAAAATTTATGGTTGAATCTCAGGTTATGGTGGATGCAGATGCACCTAGCCCTAGCAACCCTGTCTTGAGGGAATACCTTCACTGGT

GAATGAGTTATCTTTGCTTCTGTAGTCTCCCAATTGCTTCTGTTTTTAATTTCTATTAACAAGGAATACCTTCACTGATATTGATTTTTT
TTAAAAGGCAAATGAATTATTATTAATTAAGAAGAAAACAGGATCAAGGTCACAAGGAGTGTGCCACGAACCATGCATCAATATTA
CAAATCATCGGGGTCAAGAAATTAAAACATATAGCTTTCCATAAAAGCAGGATACACCCCGTAACCAAGCTTATATTGATTATAATTT
GTATCCAAAATTTATATAGCAGTAGAGCCTAAATATGCTCAAATTTTATAATTTTTTCGTGAACTTGTTTATTGACATATATTATATTCGT
TTGTTATCAATCTTTCTCTCTTTAACAATGACAGGATGGTGACAGATATTCCAGCTACCACAAATGCAAGCTTTGGTAAGTACAAATA
TTCTAAGTGTTGTGTATATGCTTAAGTAGCCGTGGGATAAAAATAACATGTTAAAGTTTCACATCAATTCAAATTAAAGATATGACCA
AAATAAATTATATAAGTGAGAAGTAATCCTAATCTTATAAATTTAAGATTGAGTTAGATTTAAATTCACTTTTTATAATTATATATATATAT
AGACACACATGACATGAGACATTAATATTGATGCACAGGGAGAGAGGTTGTGTTTTATGAGAGCCCGAACCCTTCAGTAGGGATTC
ATCGAATCGTGTTCGTATTGTTCCAGCAATTGGGCAGAGACACTGTCATCACCCCAGAATGGCGCCATAATTTCAATTCCAGAAACT
TTGCTGAAATTAATAACCTTGCACCTGTTGCAGCAGCTTATGCCAACTGCCAAAGAGAGCGTGGTTGCGGTGGAAGGAGATATTAA
CAAGTTCAATTAATGAAGATTCTTGGATGCCTTACTCCAGCTTATACTTTAGAGAAATAAAGACAGAAGAGAGAAAAATATATATAGT
ACCTTAAGTAATATGATGTAATAAAAGGAAAGATAAATAATGATAATAATAATAATGATAATAATAATAATAGATGCTTAGTGTAATAG
AATAGTAGCACTATCATCAAATAAGTTAGAAGAGTAATGGAGAAAAAGAATAAGTTAGAAGTTAATAAAATCTCTCCTTTTTTAAAT
AATAATTTAGAGAGAAGGTACCATCTTGCAATGCTAGAGCATGCCATTCAGGAACTTCTTCAACAGATTATGGTAGCAACGGAAAA
AAAAATACAAAAAGTTCATGCATGGTTCATACATACTACAGGGATCTCATCCTGTATTATTGTAATGTACGAACTGTTATTCATTTTAA
ACAACGTTTGCTTCAATAAATTACATACAATATTATTAACATTTTACCCTTTCAATTTTTTTATGATGTCCTTTCTTTTCTGGCTATCCTT
GTTTTA

Genome sequence of *GmFT5a* in ZGDD   Location: Chr16: 4135885-4137742

CCAAACACAAACATCATAGTATATAGTATCTATCTAAAGTAAATAATCATGGCACGGGAGAACCCTCTTGTTATTGGTGGTGTGATTG
GGGATGTTCTCAACCCTTTTACAAGCTCCGTTTCTTTGACTGTTTCAATCAATAATAGGGCGATTAGCAATGGCTTGGAACTCAGGC
CCTCTCAAGTTGTTAATCGCCCTAGGGTTACTGTTGGTGGTGAAGACCTAAGGACCTTCTACACTCTGGTAAGTAATTAAGTAGTATA
TATAGTGGAAATTAATCAATTGCTAGCTAGCGAGTACAAATTAGTCTTAATAAATAAGTTATTAACAGCTAGTATGCATGCTATATATAT
AAAAATTTATGGTTGAATCTCAGGTTATGGTGGATGCAGATGCACCTAGCCCTAGCAACCCTGTCTTGAGGGAATACCTTCACTGGT
GAATGAGTTATCTTTGCTTCTGTAGTCTCCCAATTGCTTCTGTTTTTAATTTCTATTAACAAGGAATACCTTCACTGATATTGATTTTTT
TTAAAAGGCAAATGAATTATTATTAATTAAGAAGAAAACAGGATCAAGGTCACAAGGAGTGTGCCACGAACCATGCATCAATATTA
CAAATCATCGGGGTCAAGAAATTAAAACATATAGCTTTCCATAAAAGCAGGATACACCCCGTAACCAAGCTTATATTGATTATAATTT
GTATCCAAAATTTATATAGCAGTAGAGCCTAAATATGCTCAAATTTTATAATTTTTTCGTGAACTTGTTTATTGACATATATTATATTCGT
TTGTTATCAATCTTTCTCTCTTTAACAATGACAGGATGGTGACAGATATTCCAGCTACCACAAATGCAAGCTTTGGTAAGTACAAATA
TTCTAAGTGTTGTGTATATGCTTAAGTAGCCGTGGGATAAAAATAACATGTTAAAGTTTCACATCAATTCAAATTAAAGATATGACCA
AAATAAATTATATAAGTGAGAAGTAATCCTAATCTTACAAATTTAAGATTGAGTTAGATTTAAATTCACTTTTTATAATTATATATATATA
TAGACACACATGACATGAGACATTAATATTGATGCACAGGGAGAGAGGTTGTGTTTTATGAGAGCCCGAACCCTTCAGTAGGGATT
CATCGAATCGTGTTCGTATTGTTCCAGCAATTGGACAGAGACACTGTCATCACCCCAGAATGGCGCCATAATTTCAATTCCAGAAAC
TTTGCTGAAATTAATAACCTTGCACCTGTTGCAGCAGCTTATGCCAACTGCCAAAGAGAGCGTGGTTGCGGTGGAAGGAGATATTA
ACAAGTTCAATTAATGAAGATTCTTGGATGCCTTACTCCAGCTTATACTTTAGAGAAATAAAGACAGAAGAGAGAAAAATATATATA
GTACCTTAAGTAATATGATGTAATAAAAGGAAAGATAAATAATGATAATAATAATAATGATAATAATAATAATAGATGCTTAGTGTAATA
GAATAGTAGCACTATCATCAAATAAGTTAGAAGAGTAATGGAGAAAAAGAATAAGTTAGAAGTTAATAAAATCTCTCCTTTTTTAAA
TAATAATTTAGAGAGAAGGTACCATCTTGCAATGCTAGAGCATGCCATTCAGGAACTTCTTCAACAGATTATGGTAGCAACGGAAAA
AAAAATACAAAAAGTTCATGCATGGTTCATACATACTACAGGGATCTCATCCTGTATTATTGTAATGTACGAACTGTTATTCATTTTAA
ACAACGTTTGCTTCAATAAATTACATACAATATTATTAACATTTTACCCTTTCAATTTTTTTATGATGTCCTTTCTTTTCTGGCTATCCTT
GTTTTA
